# Cisplatin-resistant NSCLC cells induced by hypoxia transmit resistance to sensitive cells through exosomal PKM2

**DOI:** 10.7150/thno.51797

**Published:** 2021-01-01

**Authors:** Dongliang Wang, Chaoshuai Zhao, Fei Xu, Aimi Zhang, Mingming Jin, Kunchi Zhang, Liu Liu, Qian Hua, Jian Zhao, Jianjun Liu, Hao Yang, Gang Huang

**Affiliations:** 1 Department of Nuclear Medicine, Ren Ji Hospital, School of Medicine, Shanghai Jiao Tong University, Shanghai 200127, China; 2 Shanghai Key Laboratory of Molecular Imaging, Shanghai University of Medicine and Health Sciences, Shanghai 201318, China; 3 Department of Dermatology, Shanghai General Hospital, School of Medicine, Shanghai Jiao Tong University, Shanghai 200080, China; 4 Department of Nuclear Medicine, Shanghai Chest Hospital, Shanghai Jiao Tong University, Shanghai 200030, China

**Keywords:** Exosomes, NSCLC, Drug-resistance, PKM2, CAFs

## Abstract

Hypoxia is commonly observed in solid tumors and contributes to the resistance of DNA damage drugs. However, the mechanisms behind this resistance are still unclear. In this study, we aimed to explore the effects of hypoxia-induced exosomes on non-small cell lung cancer (NSCLC).

**Methods:** NSCLC cells were subjected to either normoxic or hypoxic conditions to assess cell survival and changes in the expression levels of key proteins. Comparative proteomics were performed to identify exosomal PKM2 in normoxic or hypoxic cisplatin-resistant NSCLC cells-derived exosomes. Functions of hypoxia induced-exosomal PKM2 in promoting cisplatin resistance to NSCLC cells were evaluated both *in vitro* and *in vivo* experiments and the molecular mechanisms of hypoxia induced-exosomal PKM2 were demonstrated using flow cytometry, immunoblotting, oxidative stress detection and histological examination. A series of *in vitro* experiments were performed to evaluate the function of hypoxia-induced exosomes on cancer-associated fibroblasts (CAFs).

**Results:** Hypoxia exacerbated the cisplatin resistance in lung cancer cells due to the increased expression of PKM2 that was observed in the exosomes secreted by hypoxic cisplatin-resistance cells. We identified that hypoxia-induced exosomal PKM2 transmitted cisplatin-resistance to sensitive NSCLC cells *in vitro* and *in vivo*. Mechanistically, hypoxia-induced exosomal PKM2 promoted glycolysis in NSCLC cells to produce reductive metabolites, which may neutralize reactive oxygen species (ROS) induced by cisplatin. Additionally, hypoxia-induced exosomal PKM2 inhibited apoptosis in a PKM2-BCL2-dependent manner. Moreover, hypoxia-induced exosomal PKM2 reprogrammed CAFs to create an acidic microenvironment promoting NSCLC cells proliferation and cisplatin resistance.

**Conclusions:** Our findings revealed that hypoxia-induced exosomes transmit cisplatin resistance to sensitive NSCLC cells by delivering PKM2. Exosomal PKM2 may serve as a promising biomarker and therapeutic target for cisplatin resistance in NSCLC.

## Introduction

Lung cancer is one of the deadliest cancers affecting both men and women. Both the incidence and mortality rates of lung cancer have significantly improved over the last 50 years, however more optimal treatment and outcomes for this disease are needed 1, 2. Approximately 85% of lung cancer patients are with the non-small-cell lung cancer (NSCLC) histological subtype 3. Chemotherapy is the main treatment for lung cancer and platinum based dual therapy is the standard treatment for advanced stage patients 4. However, clinical drug resistance is still a challenge, greatly hindering treatment success. Consequently, a more comprehensive understanding of the mechanisms of drug resistance is necessary to fully combat this disease.

Some studies have reported that exosomes play an essential role in the tumorigenesis of lung cancer 5, 6. Exosomes, membrane-bound vesicles produced by late endosomes, contain different biological molecules that influence cell functions by acting as messengers in the microenvironment 7, 8. A recent study showed that the delivery of EphA2 protein through exosomes enhanced gemcitabine resistance in pancreatic cancer 7. Therefore, exosomes may impact cell functions by directly transferring biomolecules to cells or by modifying the microenvironment.

Hypoxia, a common phenomenon observed in most malignant tumors, is involved in drug resistance and tumorigenesis 9. Hypoxia in tumorigenesis occurs when there is an insufficient oxygen supply caused by the irregularity of or the distance between tumor blood vessels 10. In response to hypoxia, cancer cells adapt to the hypoxic environment through a variety of cellular mechanisms, allowing them to survive 11. One important change that occurs in a hypoxic environment is the alteration of glucose metabolism, which is the transformation from oxidative phosphorylation to glycolysis to meet the energy requirements of tumor cells. Reprogramming of tumor metabolism, termed the Warburg effect, is considered as a hallmark of cancer 12. PKM2, an important regulator of the Warburg effect, catalyzes the synthesis of pyruvate from phosphoenolpyruvate (PEP) to promote anaerobic glycolysis, which allows tumor cells to thrive. A view that PKM2 is beneficial to cancer progression is that it acts as a transcriptional co-activator of HIF-1α and regulates the metabolic reprogramming of cancer cells under hypoxic conditions 13. Another study suggested that Histone H3 is phosphorylated by PKM2 after EGF receptor activation, which is essential for tumorigenesis and promoting gene transcription 14. These studies indicate that PKM2 not only plays a role in aerobic glycolysis, but also regulates gene transcription.

Many studies have explored the effects of hypoxia on the tumor environment. However, little is known about the mechanisms of this environment as well as hypoxia-induced exosomes related to cisplatin resistance in NSCLC. In the study presented here, functional assays demonstrate the role of hypoxia in promoting cisplatin resistance through increasing the expression of PKM2 and enhancing glycolysis. Mechanistically, hypoxia-induced exosomes were found to play a role in drug resistance. In this work, we mainly focus on hypoxia-induced exosomes directly leading to cisplatin resistance in lung cancer by transferring PKM2 to tumor cells. We also focus on how hypoxia-induced exosomes indirectly promote cisplatin resistance through reprogramming cancer-associated fibroblasts (CAFs). The change of CAF metabolism after reprogramming regulates cisplatin resistance of adjacent tumor cells. Results reveal a novel mechanism of cisplatin resistance in NSCLC and suggest a promising metabolic blocker for antitumor therapy.

## Materials and Methods

### Cell culture

A549, H1299 and PC9 cell lines were purchased from the American Type Culture Collection (ATCC) and cultured in DMEM (containing 4.5 g/L D-Glucose and no sodium pyruvate, GIBCO, Grand Island, NY, USA) supplemented with 100 μg /mL streptomycin, 10% fetal bovine serum and 100 U/mL penicillin (GIBCO, Grand Island, NY, USA). Based on previous studies, the A549 cell line, which is sensitive to cisplatin (A549/SEN), was exposed to cisplatin at increasing concentrations for 10-months to establish a cisplatin-resistant A549 cell line (A549/CR) 15-17. CAFs were isolated from the tumor samples of two patients enrolled in Shanghai Chest Hospital. Tumor tissues were washed in PBS, cut into 3-4 mm pieces and digested in 1 mg/mL collagenase I (YEASEN Biotech, Shanghai, China) for 8 hours. CAFs were filtered through a 200-mesh filter of digestion solution and cultured in DMEM supplemented with 15% fetal bovine serum. All cells were cultured in a humidified atmosphere at 37 °C with 5% CO_2_ in normoxic (21% O_2_) or hypoxic (1% O_2_) environments.

### Proliferation and cytotoxicity assays

Cells were seeded into 96-well plates at a density of 10^4^ cells/well and cultured in a 37 °C incubator. Cells were treated with cisplatin, PKM2 inhibitor (PKM2-IN, Selleck Chemicals, Shanghai, China), pyruvate or lactate (Sigma Chemicals, MO, USA) at different concentrations. Cell Counting Kit-8 (CCK-8, Bimake, Shanghai, China) was used to measure relative cell viability 48h after transfection based on the manufacturer's protocol. Each sample was measured as an optical density (OD) value at 450 nm and cell viability was calculated as the ratio of the OD values between drug-treated and vehicle-treated cells. IC50 values of cisplatin were determined for A549/SEN and A549/CR cells using inhibition dose-response curves with variable slopes. For the colony formation assay, 500 cells were seeded in 12-well plates and cultured in an incubator containing 5% CO_2_ at 37 °C for 10 days. Next, cell colonies were washed three times with PBS before being fixed with 4% PFA and stained with 0.1% crystal violet. Colonies were counted and classified by the size as: small (<0.5 mm), medium (>0.5 mm; <1 mm) and big (>1 mm).

### Glucose uptake, lactate and extracellular pyruvate production

Cells were cultured in 12-well plates and medium was replaced with 500 μL serum-free high-glucose DMEM for 6 hours. The Lactate, Pyruvate (Jiancheng Bioengineering Institute, Nanjing, China) and Glucose Assay (GAGO20, Sigma-Aldrich, MO, USA) kits were used to measure extracellular levels of lactate, pyruvate and glucose, respectively, based on instructions developed by manufacturers. Data were normalized to total cell number for each test.

### Exosome isolation

Exosomes were obtained from conditioned medium culturing A549/SEN, A549/CR and hypoxic cisplatin-resistant A549 cells (hA549/CR). In short, supernatants were filtered using a 0.22 μm syringe filter and isolated by centrifugation at 120,000 g for 90 min (4 °C). Next, exosome pellets were collected by centrifugation at 120,000 g (4 °C) for 90 min again, followed by a wash with PBS. In the final step, exosome pellets were resuspended in PBS and stored at -80 °C. The process of exosome purification was completed using OptimaTM XPN-100 (Beckman Coulter) and the concentration of exosomes was measured using the Pierce BCA protein assay kit (Thermo Fisher Scientific).

### Liquid chromatography-tandem mass spectrometry (LC-MS/MS)

CRexo and hCRexo (each group contained 3 independent replicate samples) were ground into dry powder using liquid nitrogen and then precipitated with propanol for 2 h, followed by being digested using trypsin. The enzymolysis solution containing 100 μg protein was removed and an equal volume of 0.1% formic acid (FA) was added for acidification. The acidified enzymolysis solution was added to the Strata-x C18 column (Phenomenex, USA) three consecutive times and 0.1% FA + 5% acetonitrile was added to clean the Strata-x C18 column. This was eluted once with 1ml 0.1% FA + 80% acetonitrile. Each sample was analyzed using AB SCIEX nanoLC-MS/MS (AB SCIEX, USA). MaxQuant v1.5 software was used for label-free proteome identification and quantification. Identified proteins were quantified using a iBAQ algorithm 18.

### Fluorescence labeling and intracellular immunofluorescence

Exosomes resuspended in PBS were labelled with green fluorescent membrane dye PKH67 (PKH67GL, Sigma-Aldrich, St. Louis, MO, USA). Cells were treated with 40 μg/mL exosomes labelled PKH67 or PBS solution in six-well plates at 37 °C for 24 hours. Then, slides were washed three times using PBS, fixed with 4% formaldehyde for 20 min, followed by three additional PBS washes. Next, actin protein was labeled by iFluor™ 555 phalloidin (YEASEN, Shanghai, China) and cell nuclei were stained by DAPI fluorescent stain (D9542, Sigma-Aldrich, USA). Intracellular immunofluorescence was used to examine PKM2 expression. After fixing, cells on slides were incubated overnight with PKM2 antibody. Cell nuclei were stained by DAPI fluorescent stain. All images were taken using the Olympus FluoView FV1000 confocal microscope (Olympus, London, England).

### Oxidative stress detection

Following cisplatin treatment, cells cocultured with exosomes were lysed using ultrasound. Nicotinamide adenine dinucleotide (NADH) and glutathione (GSH) levels were measured using the NAD/NADH Quantitation Kit (MAK037, Sigma-Aldrich, USA) and Reductive GSH Content Assay Kit (Solarbio, Beijing, China) respectively, based on instructions provided by the manufacturer. Reactive oxygen species (ROS) levels were measured using a fluorescent 2′, 7′-dichlorofluorescin diacetate (DCFH-DA) assay as described by the manufacturers (Jiancheng, Nanjing, China). All data were normalized to total cell number.

### Apoptosis assay

Apoptosis was analyzed using the FITC Annexin V Apoptosis Detection Kit I (BD Pharmingen, CA, USA). Briefly, 10^6^ cells were washed using PBS and treated with Annexin V Binding Buffer followed by resuspension. Next, samples were incubated with 5 μl Annexin V-FITC and 5 μl propidium iodide (PI) at room temperature for 15 minutes without light. Data were quantified using flow cytometry (NovoCyte, Agilent Technologies, CA, USA) and the proportion of cells in different stages were analyzed using FlowJo VX software.

### Co-cultivation of heterogeneous cells

CAFs were first co-cultured in medium containing 40 μg/mL exosomes for 48 h in a 0.1 μM polyethylene terephthalate (PET) membrane (Jet Bio-Filtration, Guangzhou, China) insert. A549/SEN cells were seeded into 12-well plates and then the insert containing exosome-pretreated CAFs was placed in a 12-well plate. Biological properties of A549/SEN co-cultured with CAFs were subsequently tested.

### Statistical analysis

Statistical analysis was performed using GraphPad Prism 7.0 (Graphpad Software, Inc., USA) and SPSS 20.0 software (SPSS, Inc., Chicago, IL). Results were presented as mean ± S.D. Statistically significant differences comparing three or more groups were analyzed using one-way analysis of variance (ANOVA) followed by the Bonferroni post-hoc test. P values <0.05 were considered as statistically significant.

## Results

### Hypoxia exacerbates cisplatin resistance in lung cancer cells

**Figure 1:**
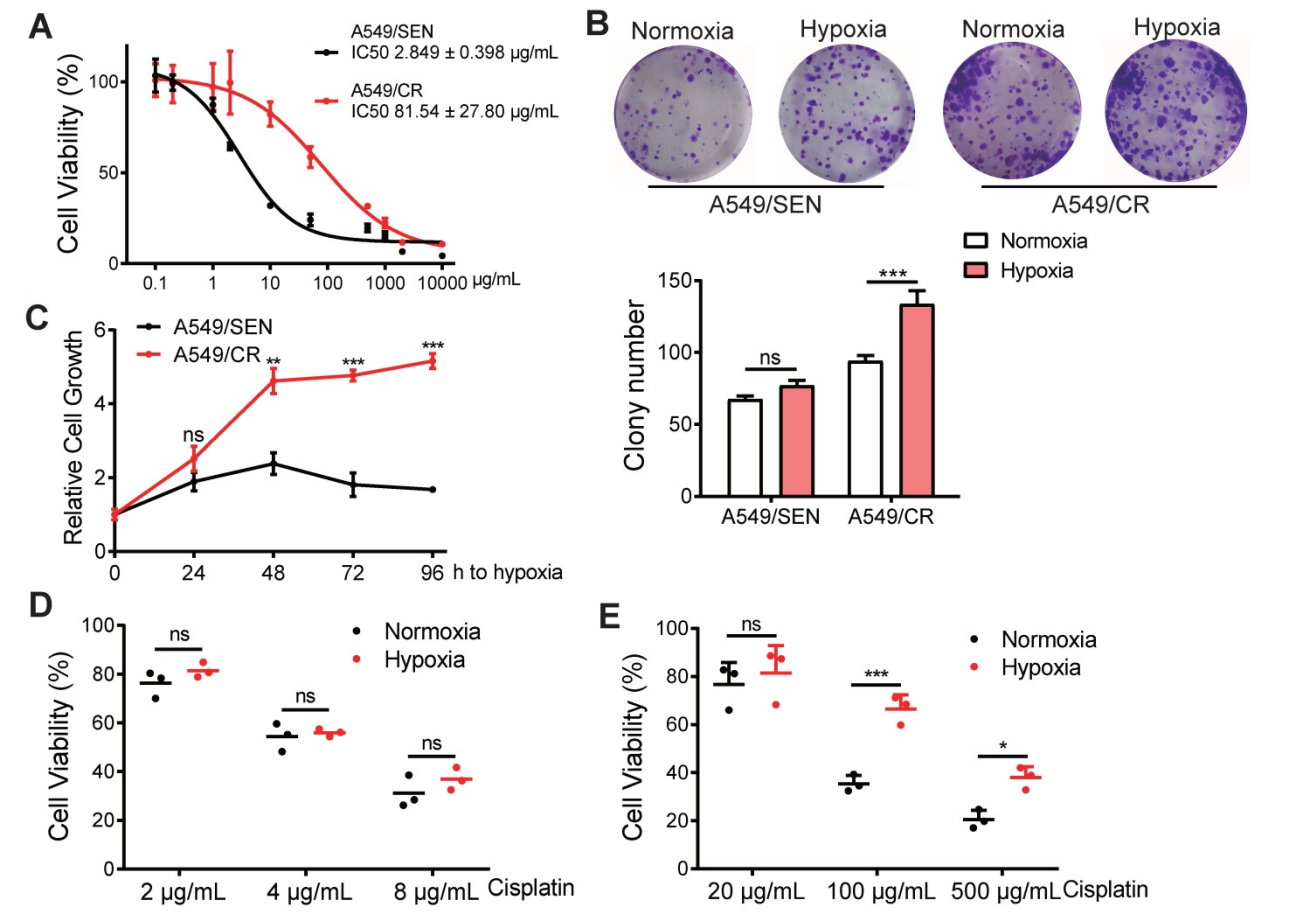
**An hypoxic environment increases cisplatin-resistance in NSCLC cells. (A)** IC50 values of A549/SEN and A549/CR cells treated with cisplatin for 48 h. The IC50 values of the two cell lines to cisplatin were statistically different (p<0.001).** (B)** Relative colony numbers (bottom) and representative images (top) of A549/SEN and A549/CR cells. Cells were cultured under normoxic and hypoxic conditions. **(C)** CCK8 assays of hypoxia tolerance of A549/SEN and A549/CR cells under hypoxic conditions. **(D)** Cell viability analysis of A549/SEN cells treated in normoxic or conditions and further treated with different concentrations of cisplatin for 48 h. **(E)** Cell viability analysis of A549/CR cells treated in normoxic or hypoxic conditions and further treated with different concentrations of cisplatin for 48 h. Data are shown as mean ± S.D. including three independent experiments. *p < 0.05; ***p < 0.001; ns, no significance.

Microenvironment hypoxia is one of the most important characteristics of a tumor. To investigate the function of hypoxia on cisplatin-resistance, we screened the cisplatin-resistant A549 cell line (A549/CR) using IC50 curve tests to reveal that A549/CR cells were more resistant to cisplatin compared with the A549/SEN sensitive cell line (Figure 1A). To elucidate the function of hypoxia on NSCLC cell behavior, colony formation assays were used to measure cell viability of both A549/SEN and A549/CR cells cultured in normoxic and hypoxia conditions. Results showed that the proliferation of A549/SEN cells under normal and hypoxic conditions did not differ. However, the number of clones of A549/CR cells under hypoxic conditions was significantly higher than that under normoxic conditions and there was no significant difference in the colony size of A549/CR cells under hypoxia and normoxia conditions (Figure 1B and Figure S1A). Meanwhile, the hypoxia tolerance of A549/CR cells was higher than for the tolerance of A549/SEN cells (Figure 1C), indicating that cisplatin-resistant cells were more common in the hypoxic environment of solid tumor of NSCLC. Next, we cultured A549/SEN and A549/CR cells in normoxic and hypoxic environments, and added cisplatin. Interestingly, we found hypoxia further promoted cisplatin-resistance of A549/CR cells and this was not observed for A549/SEN cells (Figure 1D-E). These results demonstrated that cisplatin resistance of NSCLC cells increased in a hypoxic environment.

### PKM2 mediates hypoxia-induced cisplatin resistance

**Figure 2:**
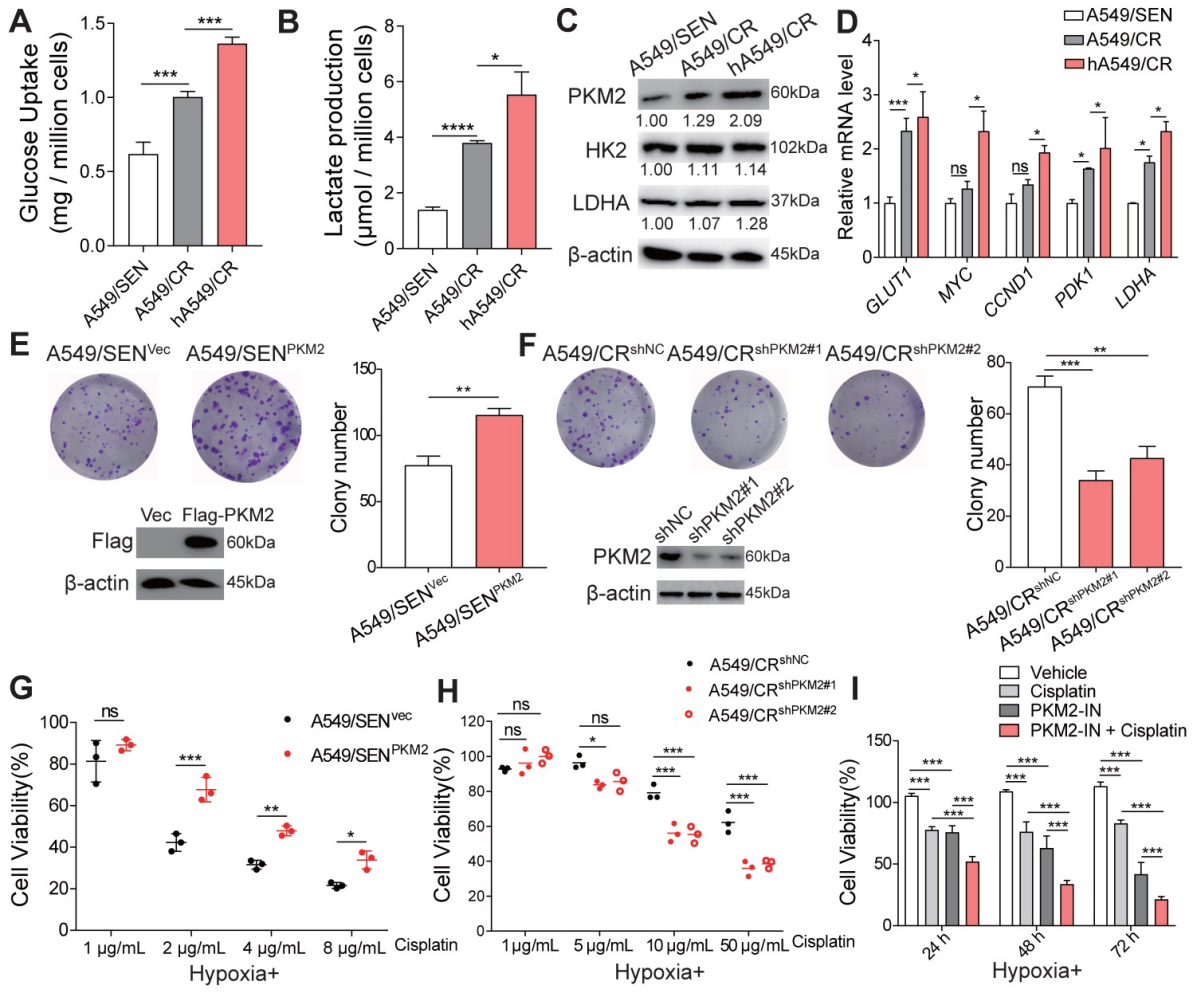
** Role of PKM2 in hypoxia-induced cisplatin resistance. (A-B)** A549/SEN cells were cultured under normoxic conditions for 24 h. A549/CR cells were cultured under normoxic or hypoxic conditions for 24 h and then serum-free medium was used for glucose uptake assays (A) and lactate production assays (B). **(C)** Immunoblotting for PKM2, HK2 and LDHA proteins in treated cells. **(D)** qRT-PCR assay for* GLUT1*, *MYC*, *CCND1*, *PDK1* and *LDHA* mRNA levels in treated cells. **(E-F)** Relative colony numbers and representative images of A549/SEN cells (E) transfected with vector (A549/SEN^vec^) or Flag-PKM2 (A549/SEN^PKM2^) treated with 2 μg/mL cisplatin and A549/CR cells (F) treated with shNC lentivirus (A549/CR^shNC^) or two different shPKM2 lentiviruses (A549/CR^shPKM2#1^ and A549/CR^shPKM2#2^) treated with 20 μg/mL cisplatin. Immunoblotting for Flag (E) or PKM2 (F) in treated cells. **(G-H)** Cell viability of A549/SEN^vec^ and A549/SEN^PKM2^ cells (G) A549/CR^shNC^, A549/CR^shPKM2#1^ or A549/CR^shPKM2#2^ cells (H) treated with different concentrations of cisplatin for 48 h under hypoxic conditions. **(I)** Cell viability of A549/CR cells treated with 20 μg/mL cisplatin, 2 μM PKM2-inhibitor (PKM2-IN) or a combination of cisplatin and PKM2-IN. Data are shown as mean ± S.D. including three independent experiments. *p < 0.05; **p < 0.01; ***p < 0.001; ns, no significance.

Since previous studies have shown that anaerobic glycolysis leads to drug-resistance in tumor cells 19, we next examined glycolysis in A549/SEN, A549/CR and hypoxic cultured A549/CR (hA549/CR) cells. A549/CR cells absorbed more glucose and produced more lactic acid compared with A549/SEN cells. In addition, hypoxia further increased glycolysis in A549/CR cells (Figure 2A-B). Meanwhile, high expression levels of three key glycolytic enzymes, including HK2, LDHA and PKM2 were observed. PKM2 expression was significantly higher in A549/CR cells compared with A549/SEN cells and hypoxia further amplified its expression in A549/CR cells (Figure 2C and Figure S1B). However, there were no significant differences observed in HK2 and LDHA expression levels between the three groups (Figure 2C). In addition, hypoxic conditions enhanced the activity of PKM2 in A549/CR cells (Figure S1C). We also found that the PKM2-HIF-1α and PKM2-β-Catenin signaling pathways in hA549/CR cells were upregulated compared with A549/CR cells or A549/SEN cells (Figure 2D). High expression of PKM2 in hA549/CR cells combined more HIF-1α and β-catenin compared with A549/SEN or A549/CR cells were observed by co-immunoprecipitation assay (Figure S1D). Since PKM mRNA produces splice variants encoding PKM1 and PKM2 isoforms 20, we explored PKM1 expression was no difference between the three groups (Figure S1E). PKM2 levels were down-regulated using stable knockdown in A549/SEN (A549/SEN^shPKM2^), A549/CR (A549/CR^shPKM2^) and hA549/CR (hA549/CR^shPKM2^) cells (Figure S1F) and then we examined the glycolysis progression. The results showed that there was no significant difference in the glucose uptake and lactate production (Figure S2A-B) among A549/SEN^shPKM2^, A549/CR^shPKM2^ and hA549/CR^shPKM2^ cells. This indicated that PKM2 is a critical mediator in the regulation of glycolysis. Next, to determine whether PKM2 regulates cisplatin resistance in NSCLC cells, PKM2 was overexpressed in A549/SEN cells (A549/SEN^PKM2^) and PC9 cells (PC9^ PKM2^) using a FLAG-tagged PKM2 plasmid. In addition, PKM2 levels were repressed using stable knockdown in A549/CR cells (A549/CR^shPKM2^). Results showed PKM2 overexpression increased the growth of A549/SEN and PC9 cells (Figure 2E and Figure S2C), whereas its knockdown decreased A549/CR cell growth (Figure 2F) when exposed to cisplatin treatment. Under hypoxic conditions, PKM2 overexpression increased the resistance of A549/SEN and PC9 cells to cisplatin at various concentrations (Figure 2G and Figure S2D), while PKM2 knockdown rescued the sensitivity of A549/CR to cisplatin (Figure 2H). Using a PKM2 specific inhibitor 21, we found that a combination of cisplatin and the PKM2 inhibitor showed stronger effects on proliferation inhibition compared to each single agent alone in hA549/CR cells (Figure 2I). Collectively, these results demonstrated that PKM2 promotes cisplatin resistance in NSCLC cells by increasing glycolysis that is enhanced by hypoxia.

### Identification of exosomal PKM2 in hypoxic cisplatin-resistant cells

**Figure 3:**
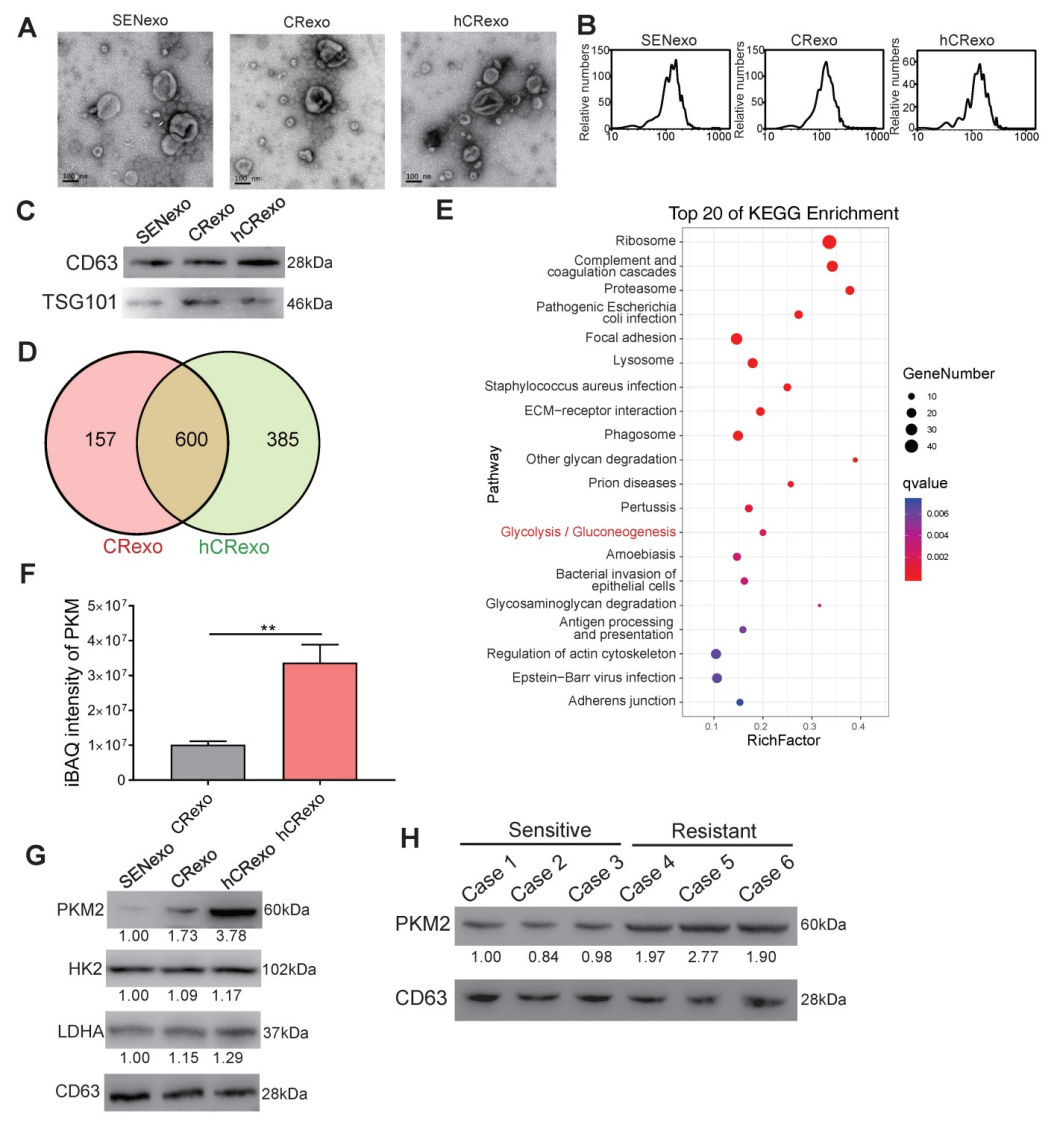
** Characterization of isolated exosomes and exosomal proteins. (A-C)** Identification of exosomes derived from A549/SEN, A549/CR and hA549/CR cells by TEM (A), NTA (B) and immunoblotting (C). **(D)** Venn diagram of identified proteins between CRexo and hCRexo from proteomics analysis by LC-MS/MS. **(E)** KEGG enrichment analysis of differential proteins in CRexo and hCRexo. **(F)** iBAQ intensity of PKM by LC-MS/MS was performed. **p < 0.01. (**G**) Immunoblotting for PKM2, HK2 and LDHA proteins in 10 μg of SENexo, CRexo and hCRexo. (**H**) Immunoblotting for PKM2 proteins in exosomes derived from cisplatin-resistant and cisplatin-sensitive lung cancer patients.

We next sought to determine whether PKM2 participated in cell-to-cell communication and cisplatin-resistance transmission through exosomes. Exosomes were isolated from A549/SEN, A549/CR and hA549/CR cells using ultracentrifugation. The morphology of the exosomes was observed by transmission electron microscopy (Figure 3A). As shown in figure 3B, the diameter of exosomes shown by light scattering studies ranged from 50-150nm. Western blotting revealed that the exosomes were enriched with the exosomal markers CD63 and TSG101 (Figure 3C), indicating that exosomes were properly isolated. To determine the differences in exosomal proteins derived from cisplatin-resistant cells in normoxic and hypoxic cultures, LC-MS/MS was used to obtain protein expression profiles of exosomes derived from A549/CR (CRexo) and exosomes derived from hA549/CR (hCRexo). Among the identified exosomal proteins, 157 proteins were specifically expressed in CRexo and 385 were expressed in hCRexo (Figure 3D). Using the iBAQ algorithm for absolute quantification of proteins, 504 proteins were found to be differentially expressed between the two groups (339 were highly expressed in hCRexo and 165 were highly expressed in CRexo, Supplementary Table S1 and S2). KEGG enrichment analysis (Figure 3E) and GO functional classification (Figure S3A) of differential proteins showed that glycolysis and related metabolic pathways were significantly associated with hypoxia-induced cisplatin resistance. Among the highly expressed proteins in hCRexo, proteins that regulate glucose metabolism, including PKM2, were listed (Figure S3B). The significant high-expressed proteins in CRexo that are involved in cell metabolism are were listed (Figure S3C). Through absolute protein quantification (Figure 3F) by MS and western blotting (Figure 3G and Figure S3D), high expression of PKM2 in hCRexo was determined. In addition, we found that PKM2 in the serum exosomes from drug-resistant patients was significantly higher than that in the exosomes of sensitive patient (Figure 3H), which revealed that our conclusion was also confirmed in clinical samples.

### Exosomes from hypoxic resistant cells deliver PKM2 to sensitive cells to transmit cisplatin resistance

**Figure 4:**
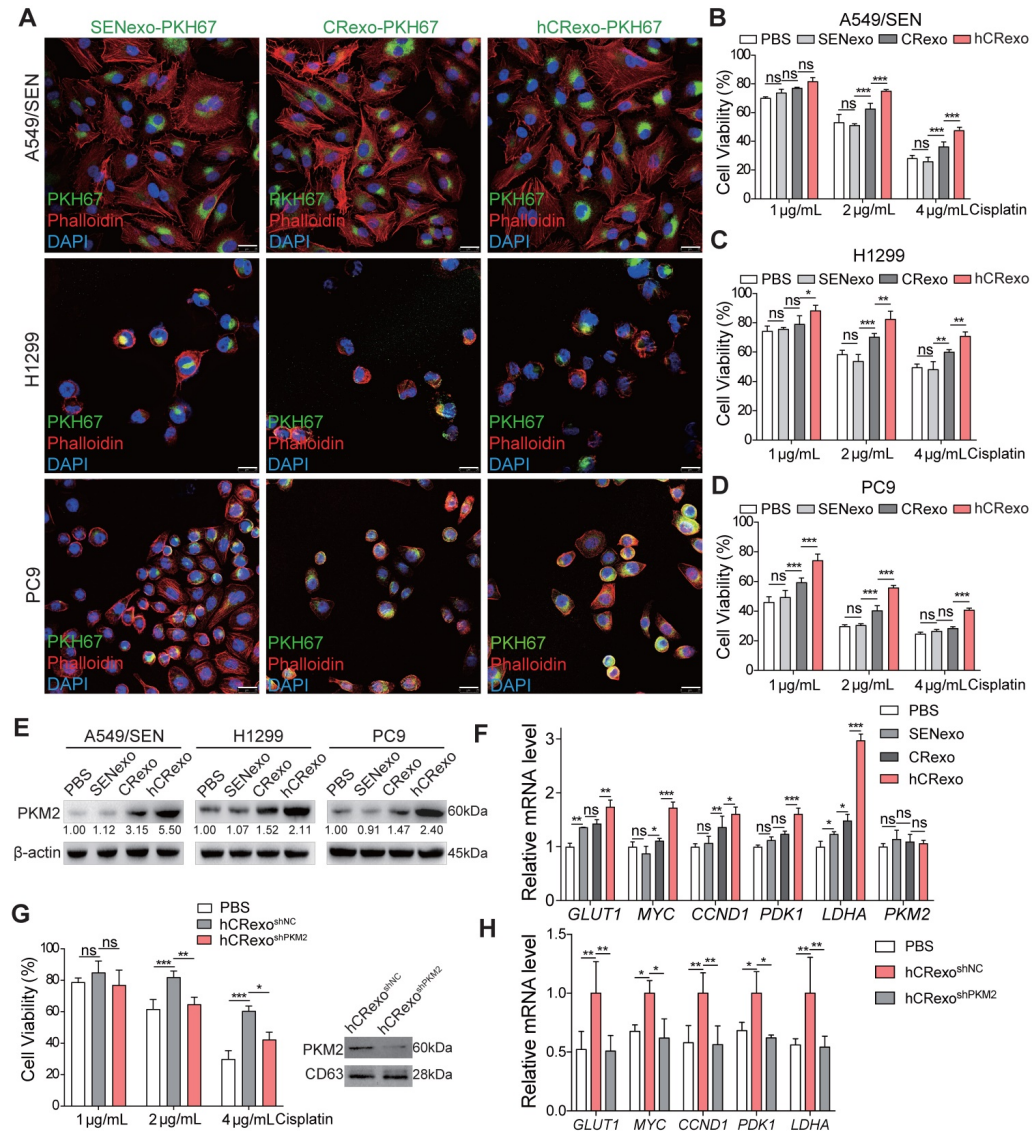
** Exosomal PKM2 transmits cisplatin-resistance to sensitive NSCLC cells. (A)** Fluorescence images of A549/SEN, H1299 and PC9 cells treated with PKH67-stained SENexo, CRexo and hCRexo (green) and then stained with phalloidin (red) and DAPI (blue) for visualization of exosomes uptake. Scale bar, 25 μm. **(B-D)** Cell viability of A549/SEN (B), H1299 (C) and PC9 cells (D) treated with 40 μg/mL SENexo, CRexo and hCRexo for 48 h, following being treated with cisplatin for 48 h. **(E)** Expression of PKM2 in treated cells. **(F)** qRT-PCR assay for *GLUT1*, *MYC*, *CCND1*, *PDK1*, *LDHA* and *PKM2* mRNA levels in treated cells. **(G)** CCK8 assay (left) for A549/SEN cells cocultured with exosomes isolated from hA549/CR cells treated with shNC lentivirus (hCRexo^shNC^) or shPKM2#1 lentivirus (hCRexo^shPKM2^). Immunoblotting (right) for PKM2 in exosomes. **(H)** *GLUT1*, *MYC*, *CCND1*, *PDK1* and *LDHA* mRNA levels in A549/SEN cells treated with exosomes. Data are shown as mean ± S.D. including three independent experiments. *p < 0.05; **p < 0.01; ***p < 0.001; ns, no significance.

It is known that exosomes affect tumor resistance to various drugs 22, 23, which led us to explore whether hA549/CR cell derived-exosomes alter cisplatin efficacy in sensitive cells. First, green fluorescent dye PKH67 labeled exosomes were incorporated into A549, H1299 and PC9 cells (Figure 4A) to confirm that cisplatin-sensitive cells effectively incorporated SENexo, CRexo and hCRexo. Next, we determined the appropriate treatment concentration of exosomes (Figure S4A) and evaluated cisplatin resistance in NSCLC cells. Compared with SENexo or CRexo, hCRexo enhanced cisplatin resistance of A549 (Figure 4B and Figure S4B), H1299 (Figure 4C and Figure S4C) and PC9 (Figure 4D and Figure S4D) cells. Meanwhile, we found that hCRexo treatment also increased PKM2 expression in cisplatin-sensitive cells (Figure 4E), while *PKM2* mRNA levels did not change in cisplatin-sensitive cells treated with hCRexo (Figure 4F). This suggested that hCRexo directly transferred PKM2 to sensitive cells, not influencing *PKM2* mRNA levels. Sensitive A549 cells that absorbed hCRexo highly expressing PKM2 exhibited increased PKM2-dependent non-metabolic transcriptional activity (Figure 4F). Co-immunoprecipitation assay showed that PKM2 from hCRexo interactd with HIF-1α or β-catenin in A549/SEN cells as detected in nuclear lysates, which revealed that the PKM2 that transferred into the nucleus to activate HIF-1α and β-catenin pathways by recruiting them (Figure S4E-F). To determine whether exosomal PKM2 induced cisplatin-resistance in sensitive cells, cell viability of A549/SEN cells under cisplatin exposure was examined. Treated exosomes were derived from hA549CR^shPKM2^ (hCRexo^shPKM2^) and control exosomes (hCRexo^shNC^) cells.

A549/SEN cells with hCRexo^shPKM2^ showed rescued sensitivity to cisplatin (Figure 4G) and decreased PKM2-dependent transcriptional activity (Figure 4H). In addition, we collected exosomes from hA549/CR cells with PKM2 inhibitor treatment (hCRexo^PKM2-In^) and found the hCRexo^PKM2-In^ failed to transmit cisplatin resistance to A549/SEN cells (Figure S4G). These results indicated that hCRexo conferred cisplatin-resistance to sensitive cells by delivering PKM2 protein.

### Hypoxic resistant cell derived-exosomes inhibit cisplatin-induced apoptosis by increasing metabolites

**Figure 5:**
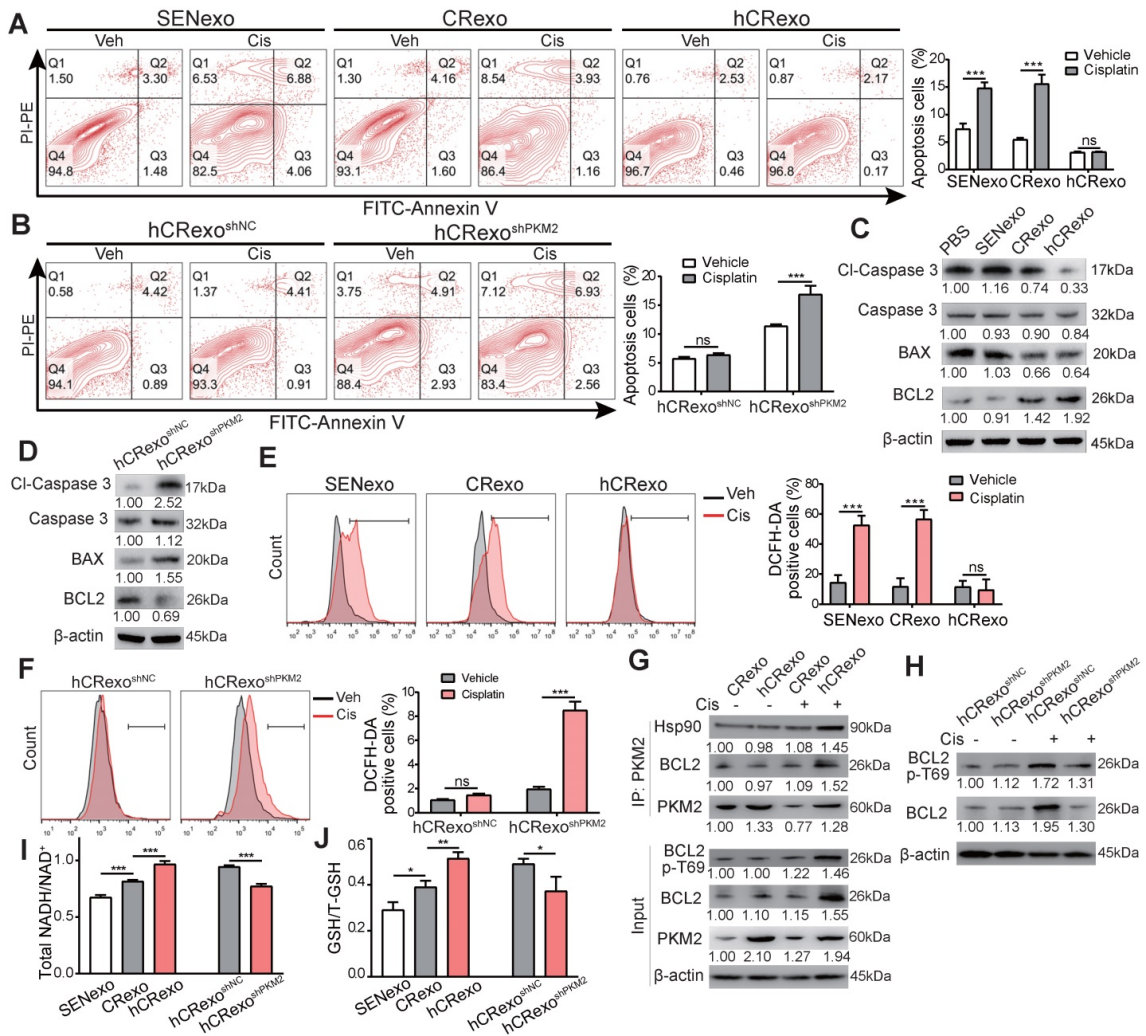
** hCRexo inhibits cisplatin-induced apoptosis through PKM2. (A-B)** Representative images (left) and proportion of apoptosis cells (right) for A549/SEN cells treated with 4 μg/mL cisplatin for 48 h after treatment with 40 μg/mL exosomes. A549/SEN cells were treated with SENexo, CRexo, hCRexo (A), hCRexo^shNC^ or hCRexo^shPKM2^ (B). **(C-D)** Immunoblotting for apoptosis-related proteins including cleaved caspase-3, caspase-3, BAX and BCL2 in -treated cells. **(E-F)** The proportion of DCFH-DA positive cells intreated cells using flow cytometry and counting. **(G)** A549/SEN cells cocultured with 40 μg/mL CRexo or hCRexo were treated with 4 μg/mL cisplatin (or vehicle). PKM2 proteins were immunoprecipitated and the interacting proteins were detected using immunoblotting. **(H)** Immunoblotting for BCL2, BCL2 p-T69 for A549/SEN cells cocultured with hCRexo^shNC^ or hCRexo^shPKM2^ treated with 4 μg/mL cisplatin (or vehicle). **(I-J)** The total NADH/ NAD^+^ (I) and GSH/T-GSH (J) in A549/SEN cells treated with 40 μg/mL SENexo, CRexo, hCRexo, hCRexo^shNC^ or hCRexo^shPKM2^. Data are shown as mean ± S.D. including three independent experiments. *p < 0.05; **p < 0.01; ***p < 0.001; ns, no significance.

To investigate the mechanisms related to how hCRexo promoted cisplatin resistance in NSCLC cells, we first examined apoptosis of A549/SEN cells co-cultured with SENexo, CRexo and hCRexo under cisplatin treatment. A549/SEN cells treated with SENexo and CRexo exhibited apoptosis induced by cisplatin, while the hCRexo group showed no significant differences in apoptosis despite cisplatin treatment (Figure 5A). Meanwhile, hCRexo treatment significantly alleviated cisplatin-induced nuclear irregularity, condensation and fragmentation (Figure S5), indicating that hCRexo inhibited cisplatin-induced apoptosis. However, hCRexo^shPKM2^ lost the ability to inhibit apoptosis in A549/SEN cells treated with cisplatin (Figure 5B). A549/SEN cells treated with hCRexo after cisplatin treatment exhibited increased BCL2 expression and decreased cleaved caspase 3 levels, while hCRexo^shPKM2^ reversed this effect (Figure 5C-D). Considering that intracellular reactive oxygen species (ROS) are implicated in apoptosis and chemotherapy effects 24, 25, we analyzed how hCRexo influenced intracellular ROS levels in A549/SEN cells after cisplatin treatment. Compared with CRexo and SENexo, hCRexo significantly reduced the number of ROS-positive cells induced by cisplatin (Figure 5E). hCRexo-mediated inhibition of ROS levels was remarkably attenuated in hCRexo with PKM2 downregulation (Figure 5F). Therefore, delivery of PKM2 by hCRexo clears cisplatin-induced intracellular ROS in sensitive cells.

PKM2 recruits Hsp90, phosphorylates BCL2 at threonine 69 site (T69) and stabilizes BCL2 to inhibit ROS induced apoptosis 26. Thus, we speculated that exosomes mediated by PKM2 may use a similar mechanism. A co-immunoprecipitation assay was performed to validate both the PKM2-Hsp90 and PKM2-BCL2 interactions in A549/SEN cells treated with exosomes after cisplatin treatment. We found that hCRexo enhanced BCL2 expression and increased phosphorylation of T69 by delivering PKM2 and revealed that hCRexo promoted recruitment of PKM2 to Hsp90 and BCL2 (Figure 5G). These observations were further supported when PKM2 knockdown in hCRexo did not show an increase in BCL2 expression and activation of phosphorylation in A549/SEN cells (Figure 5H). These results showed that hCRexo inhibited ROS-mediated apoptosis by inducing the interaction between PKM2 and BCL2. GSH, maintained by NADH in its reduced state, acts as an important antioxidant and eliminates ROS 27. hCRexo increased levels of reduced NADH (Figure 5I) and GSH (Figure 5J) compared with the CRexo and SENexo groups. The contents Levels of reduced NAPH (Figure 5I) and GSH (Figure 5J) under hCRexo^shPKM2^ treatment were decreased compared with hCRexo^shNC^. Taken together, hCRexo inhibits apoptosis of NSCLC cells by promoting PKM2-dependent BCL2 activation and increases reduced NADH and GSH levels in the PKM2-mediated metabolic pathway.

### Hypoxic resistant cell-derived exosomes reprogram CAF metabolism by delivering PKM2 to promote resistance in sensitive cells

**Figure 6:**
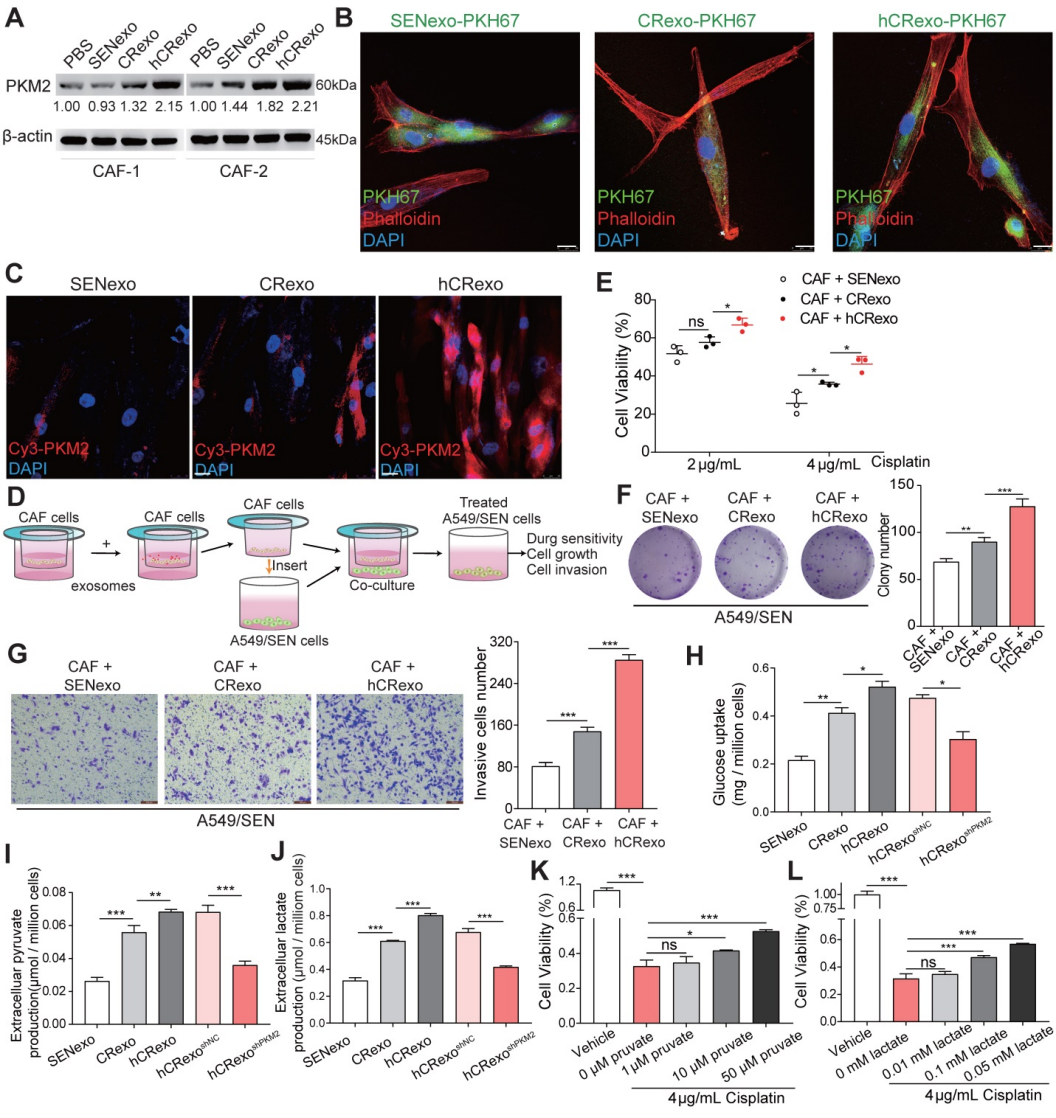
** hCRexo reprograms CAFs metabolism to promote cisplatin-resistance in sensitive NSCLC cells. (A)** Two CAF cell lines were cocultured with PBS, SENexo, CRexo or hCRexo. Immunoblotting for PKM2 in the above-treated cells. **(B)** Fluorescence confocal microscopy of CAFs after being incubated PKH67-labeled (green) SENexo, CRexo and hCRexo. Cell nuclei were stained with DAPI (blue) and cytoskeleton was stained with phalloidin (red). Scale bar, 25 μm. **(C)** Immunofluorescence images of Cy3-labeled PKM2 (red) in CAFs treated with SENexo, CRexo or hCRexo. Cell nuclei were stained with DAPI (blue). Scale bar, 25 μm. **(D)** Schematic diagram of coculturing CAFs and A549/SEN cells. CAFs were pretreated with SENexo, CRexo or hCRexo and then cocultured with A549/SEN cells following 4 μg/mL cisplatin treatment. **(E)** Cell viability of treated cells. **(F)** Relative colony numbers (right) and representative images (left) of treated cells. **(G)** Transwell assay images (left) and invasive cell numbers (right) of treated cells. **(H-J)** CAFs were treated with 40 μg/mL SENexo, CRexo and hCRexo for 48h and then treated in serum-free medium for glucose-uptake assays (H), extracellular pyruvate production assays (I) or lactate production assays (J). **(K-L)** Cell viability of A549/SEN cells treated with different concentrations of pyruvate (K) and lactate (L) following 4 μg/mL cisplatin treatment for 48h. Data are shown as mean ± S.D. including three independent experiments. *p < 0.05; **p < 0.01; ***p < 0.001; ns, no significance.

Previous studies confirmed that exosomes regulate the metabolic reprogramming of CAFs and improve the tumor malignant microenvironment 28. We confirmed whether hCRexo altered glycolysis observed in CAFs and whether it influenced cisplatin therapy in A549/SEN through CAF-related metabolic changes. Two CAFs were isolated from human lung cancer tissues and co-cultured with exosomes. The expression of PKM2 was highest in the two CAFs treated with hCRexo (Figure 6A). CAF-1 images obtained using confocal microscopy showed that exosomes were absorbed by CAFs (Figure 6B) and that PKM2 levels were increased in CAFs after hCRexo treatment (Figure 6C). Altogether, these findings suggested that hCRexo transferred PKM2 to CAFs. Furthermore, we aimed to test whether CAFs-pretreated with hCRexo functioned along with NSCLC cells. Exosomes were treated with CAFs for 48 hours and then co-cultivated with A549/SEN to analyze the behavior of A549/SEN cells (Figure 6D). CAFs-pretreated with hCRexo showed significantly increased cisplatin-resistance in A549/SEN cells compared to CAFs-pretreated with CRexo or SENexo (Figure 6E). Colony formation assays showed similar results as colony number was higher in pretreated hCRexo groups compared with groups pretreated with CRexo or SENexo (Figure 6F). Moreover, transwell assays showed that CAFs-pretreated with hCRexo significantly increased the invasion of A549/SEN cells (Figure 6G). As expected, compared with SENexo or CRexo, glucose uptake (Figure 6H), extracellular pyruvate (Figure 6I) and lactate secretion (Figure 6J) were distinctly increased in CAFs treated with hCRexo, but hCRexo^shPKM2^ reduced glycolysis in CAFs. We overexpressed FLAG-tagged PKM2 in CAFs and found that upregulation of PKM2 promoted the glycolysis in CAFs (Figure S6A-C). Similar to coculturing CAFs-pretreated with hCRexo, direct addition of lactate and pyruvate also promoted cisplatin resistance (Figure 6K-L), proliferation (Figure S6D) and invasion (Figure S6E) of A549/SEN cells. These data demonstrated that CAFs-pretreated with hCRexo promoted cisplatin resistance in A549/SEN cells induced by CAF metabolic reprogramming.

### Hypoxic resistant cell-derived exosomes promote cisplatin resistance *in vivo*

**Figure 7:**
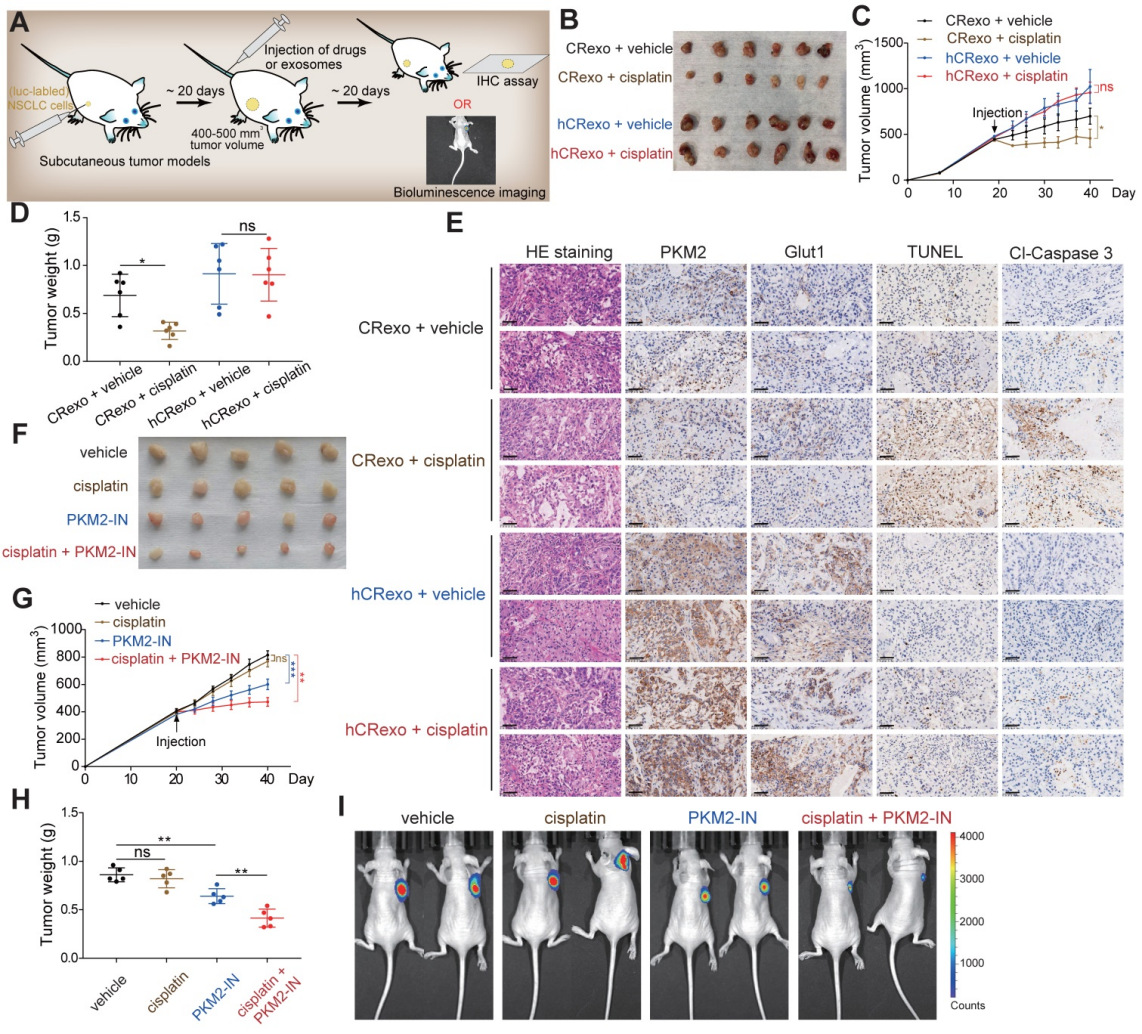
** hCRexo promotes cisplatin-resistance of NSCLC *in vivo*. (A)** Schematic diagram of the mouse xenograft model established by A549/SEN or luciferase (luc)- labeled A549/CR cells. **(B)** Image of tumors in nude mice bearing A549/SEN cells treated with CRexo and hCRexo following cisplatin treatment or treatment with control vehicle. **(C-D)** Tumor volume (C) and tumor weight (D) in (B) were measured. **(E)** Representative images of H&E staining, IHC assays for PKM2, GLUT1 and cleaved-caspase 3 (cl-caspase 3), and TUNEL detection of tumor tissues. Scale bar, 50 μm. **(F)** Image of tumors in nude mice bearing A549/CR cells expressed luciferase and then treated with cisplatin or PKM2-IN or a combination of cisplatin and PKM2-IN. **(G-H)** Tumor volume (G) and tumor weight (H) in (F) were measured. **(I)** *In vivo* bioluminescence imaging for this model (E) Data are shown as mean ± S.D. *p < 0.05; **p < 0.01; ***p < 0.001; ns, no significance.

To investigate cisplatin-resistance transmission of hCRexo *in vivo*, we established mouse subcutaneous xenograft models using A549/SEN cells. Mice with similar tumor sizes were randomly selected and divided into four groups (Figure 7A). We observed that tumor growth in the CRexo treatment group was slower than the hCRexo treatment group and that cisplatin significantly decreased tumor volume and weight in the CRexo group. However, cisplatin did not change tumor growth under hCRexo treatment (Figure 7B-D). Moreover, IHC assays showed that compared with CRexo, hCRexo treatment significantly increased BCL2, phosphorylated BCL2, PKM2 and GLUT1 expression levels in tumor tissues (Figure 7E and Figure S7). Unlike CRexo, hCRexo treatment did not increase cisplatin-induced apoptosis, as shown by the negative signal obtained by TUNEL and lower expression levels of cleaved-caspase 3 (Figure 7E). These results indicated that hCRexo promotes the expression of PKM2 and enhanced glucose metabolism *in vivo*, thereby maintaining cisplatin resistance in NSCLC. To further explore preclinical significance, we established A549/CR cells that stably expressed luciferase and subcutaneously injected them into nude mice that were randomly selected with similar tumor volumes for administration of PKM2 inhibitor (Figure 7A). Administration of PKM2 inhibitor inhibited tumor growth. Moreover, a combination of the PKM2 inhibitor and cisplatin significantly inhibited tumor growth compared to administration of each drug alone (Figure 7G-H). These results were also confirmed by luciferase real-time bioluminescence imaging (Figure 7I). In general, these *in vivo* data were consistent with our *in vitro* results indicating that hCRexo enhanced cisplatin resistance of NSCLC cells. Meanwhile, targeting PKM2 may be an effective strategy to combat cisplatin-resistant NSCLC.

## Discussion

**Figure 8:**
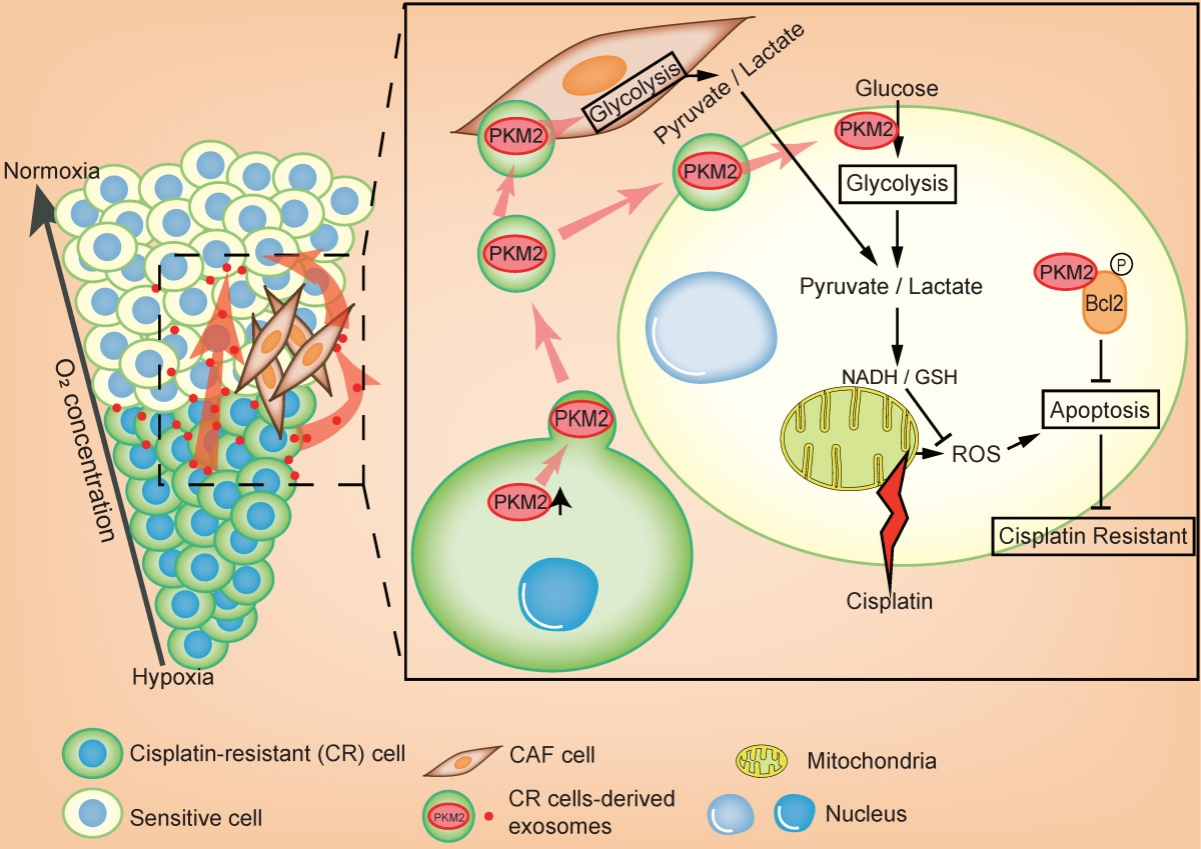
Schematic illustration of hypoxia-exosomal PKM2 in promoting cisplatin-resistance of NSCLC cells.

In this study, we revealed that exosomal PKM2 transmits NSCLC chemotherapy resistance and its mechanisms. The hypoxic area inside the tumor is usually resistant to chemotherapy and these hypoxic drug-resistant cells transmit resistance using two methods (Figure 8). First, hypoxic cisplatin-resistant cells secrete exosomes containing high levels of PKM2, which are absorbed by sensitive cells. Exosomal PKM2 also regulates glycolysis in sensitive cells to produce reduced metabolites and may neutralize ROS induced by cisplatin or to inhibit apoptosis in a PKM2-BCL2-dependent manner. Second, exosomes secreted by hypoxic cisplatin-resistant cells deliver PKM2 to CAFs in the tumor microenvironment. Metabolically reprogrammed CAFs release pyruvate and lactate, which promote proliferation, invasion and chemotherapy resistance of sensitive cells.

Multiple studies have shown that more optimal therapies are needed to treat NSCLC and that oxygen deficiency in solid tumors is one reason that current therapies are not always effective 29, 30. It is necessary to comprehensively understand the molecular mechanisms involved in drug resistance in lung cancer, which will hopefully lead to the identification of novel prognostic predictors. The study presented here demonstrated that NSCLC cells enhance drug resistance by increasing PKM2 expression in a hypoxic environment. The protein levels of other metabolic enzymes such as HK2 and LDHA were not significantly altered in hypoxia-induced drug-resistant and sensitive cells. However, we found that LDHA mRNA levels were changed in three groups (A549/SEN, A549/CR and hA549/CR). It is generally recognized that transcription and translation are independent processes containing different functional complexes and mechanisms. Even though several studies have shown that there is a certain degree of dependence between transcription and translation, recent studies on transcriptome-proteome relationships have revealed discordance of mRNA/protein expression for certain genes. For example, Yan *et al.* demonstrated that overexpression of FAT10 results in expression discordance between WISP1 protein and mRNA levels 31. Dubaisi *et al*. provided an explanation for the discordance of SULT1C4 mRNA/protein expression in prenatal livers by demonstrating that the predominant SULT1C4 transcript is a variant that produces relatively little protein 32. In our study, LDHA mRNA levels significantly different among the different cell lines, but protein expression levels remained the same. Varying degrees of mRNA and protein degradation rates may explain why the mRNA and protein levels do not correlate. This may also be caused by certain factors intervening in the LDHA mRNA translation process that require further investigation.

Exosomes are known to promote a new cell-to-cell communication method when it comes to transferring various biomolecules such as proteins, mRNAs and miRNAs to different cells 33-35. Many studies have shown that hypoxia-derived exosomes are intimately connected to transform the phenotype of recipient tumor or stromal cells by delivering proteins or genetic information 36, 37. For example, hypoxic BMSC-derived exosomes were reported to promote metastasis in lung cancer by mediating the transfer of miR-193a-3p, miR-210-3p and miR-5100 to activate STAT3 signaling-induced EMT 38. In another study, hypoxic tumor cell-derived exosomal WNT4 promoted metastatic behavior in colorectal cancer by activating β-catenin signaling 39. Moreover, a recent study showed that the hypoxic tumor cell-derived exosomal microRNAs 486-5p, 181a-5p and 30d-5p were circulating markers of high-risk, locally advanced rectal cancer (LARC) 40. However, there are limited studies illustrating that hypoxia-derived exosomes mechanistically impact the interaction between tumor cells and the microenvironment. Here, the exosomes of hypoxic resistant tumor cells were shown to act on tumor cells in normoxic conditions to establish a cisplatin-resistant phenotype as well as reprogram stromal cell CAFs.

PKM2 is a rate-limiting enzyme that catalyzes the last step of glycolysis 41. In recent years, more evidence has shown that the activity of PKM2 is closely related to the occurrence and development of tumors 41-43. However, few studies have focused on the role of PKM2 enriched exosomes in NSCLC, which our results indicate hCRexo can transfer cisplatin resistance through a PKM2-dependent mechanism. Furthermore, we observed that the expression of PKM2 was increased in exosomes under hypoxic conditions and revealed that exosomal PKM2 protein is directly transferred to tumor cells to influence malignant behavior. The high expression of PKM2 in cisplatin-resistant cells increased the production and secretion of PKM2 protein or downstream metabolites such as lactate and pyruvate acid. Our research and other study 44 suggested that lactate and pyruvate acid promoted resistance to chemotherapeutics. However, protein and metabolites are unstable and easy to decompose in the extracellular environment. We believed that the action of protein or metabolites on recipient cells lacks persistence, which may not be the main way to cause the chemoresistance. Exosomes have natural content protection properties due to the lipid bilayer membrane coating and continuously transmit PKM2 derived from cisplatin-resistant cells to sensitive cells. This may be the main mode of communication between resistant and sensitive cells.

Excessive ROS break the outer membrane of mitochondria and lead to apoptosis. Recent studies have demonstrated that somatic cells divert glycolysis to the pentose phosphate pathway (PPP) for the production of NADPH which can neutralize ROS 45. In a previous study, we found that hypoxia-induced exosomal PKM2 promoted glycolysis to produce reductive metabolites, which may neutralize ROS induced by cisplatin. Liang *et al.* reported that PKM2 inhibits apoptosis induced by oxidative stress by phosphorylating BCL2 at T69, subsequently stabilizing BCL2 26. Consistent with previous reports, our study found that exosomal PKM2 was also transported into mitochondria under oxidative stress to phosphorylate BCL2 and reduce cisplatin-induced apoptosis.

In addition to its role in tumor cells, PKM2 may also affect the tumor microenvironment to accelerate carcinogenesis through exosomes. For example, prostate cancer derived-exosomal PKM2 from the bone marrow promoted premetastatic niche formation through the up-regulation of CXCL12 in bone marrow stromal cells 46. Another study indicated that exosomal PKM2 induced monocyte-to-macrophage differentiation and remodeled the tumor microenvironment to facilitate hepatocellular carcinoma progression 47. For the first time, we showed that exosomal PKM2 induced metabolic reprogramming in CAFs, leading to drug-resistance of NSCLC cells. Recently, it has been reported that the tumor associated fibroblasts (CAFs) secrete exosomes that could regulate the microenvironment of tumors. For example, Ren *et al*. demonstrated that lncRNA H19 secreted by CAFs-exo could promote stemness and chemoresistance of colorectal cancer 48. Zhang *et al.* indicated that miR-522 in CAFs-derived exosomes promotes gastric cancer acquired chemo-resistance 49. However, the focus of our study is whether exosomes secreted by resistant tumor cells reprogrammed the metabolic state of tumor microenvironment, such as CAFs. CAFs secrete lactic acid and pyruvate to tumor cells, which enhanced the metabolic connection between CAFs and tumor cells. In this study, we revealed that hypoxia-induced exosomes delivering PKM2 transmit cisplatin resistance to sensitive NSCLC cells and exosomal PKM2 may be a promising biomarker and therapeutic target for cisplatin resistance in NSCLC. However, there are certain limitations to this study. In our study, there was a lack of sufficient patient samples to fully prove the correlation between high exosomal PKM2 expression and cisplatin resistance. Since we found that the expression of PKM2 in cisplatin-resistance cells in tumor hypoxic centers was significantly higher than in normal tumor cells, as well as some clinical evidence, we believe that exosomal PKM2 may be a potential biomarker for prediction. Although PKM2 is highly expressed in cisplatin-resistant tumors, it is also widely expressed in normal tumor tissues. In view of the more complex expression and modification in the human body, there is still a long way to go before applying these results to the clinic.

## Conclusions

Overall, this study suggests a new mechanism for the spread of drug resistance in solid tumors. Hypoxia induced-exosomes directly transmit drug resistance through a PKM2-dependent mechanism and by reprogramming CAFs to create a drug-resistant microenvironment. Exosomal PKM2 may be a potential biomarker for the clinical detection of cisplatin-resistance NSCLC, which would allow patients to be assigned to receive targeted therapy for PKM2.

### Availability of data and materials

Datasets supporting the conclusions of this study are included within the article and in the supplementary files.

## Supplementary Material

Supplementary materials and methods, figures and tables.

## Abbreviations

NSCLC: non-small Cell Lung Cancer
PKM2: pyruvate kinase isozyme type M2
CAFs: cancer-associated fibroblasts
A549/SEN: cisplatin-sensitiveA549 cell line
A549/CR: cisplatin-resistant A549 cell line
hA549/CR: hypoxic cisplatin-resistant A549 cell line
SENexo: exosomes derived from A549/SEN
CRexo: exosomes derived from A549/CR
hCRexo: exosomes derived from hA549/CR
ROS: reactive oxygen species
NADH: nicotinamide adenine dinucleotide
GSH: glutathione
LC-MS/MS: liquid chromategraphy-tandem mass spectrometry
shRNA: short hairpin RNA
BCL2: B-cell lymphoma-2
Hsp90: heat shock protein 90
DAPI: 4',6-diamidino-2-phenylindole
