# Supplementary material for: Cisplatin-resistant NSCLC cells induced by hypoxia transmit resistance to sensitive cells through exosomal PKM2
Source: Theranostics. 2021 Jan 1;11(6):2860–75. doi: 10.7150/thno.51797 (PMC7806469; doi:10.7150/thno.51797)
Supplement: Supplementary file 1 — Supplementary materials and methods, figures and tables. [file thnov11p2860s1.pdf]

Supplementary Materials and Methods

Plasmids and shRNAs

PCR-amplified human PKM2 was cloned into a pcDNA4TO-Flag vector. A549/SEN cells having 50-60% confluency were seeded into a 6-well plate and transfected with Flag-PKM2 or empty vector using Lipofectamine 3000 (Invitrogen, Carlsbad, CA, USA). For stable PKM2 knockdown, A549/CR cells were infected by lentivirus (GenePharma, Shanghai, China) with either shNC (sense 5’-CCGGTTCTCCGAACGTGTCACGTTTCAAGAGAACGTGACACGTTCGGAGAATTTTTTG-3’, antisense 5’-AATTCAAAAAATTCTCCGAACGTGTCACGTTCTCTTGAAACGTGACACGTTCGGAGAA-3’), shPKM2#1 (sense 5’-CCGGGCCCGAGGCTTCTTCAAGAAGTTCAAGAGACTTCTTGAAGAAGCCTCGGGCTTTTTTG-3’, antisense 5’-AATTCAAAAAAGCCCGAGGCTTCTTCAAGAAGTCTCTTGAACTTCTTGAAGAAGCCTCGGGC-3’), shPKM2#2 (sense 5’-CCGGAGGCAGAGGCTGCCATCTATTCAAGAGATAGATGGCAGCCTCTGCCTTTTTTTG-3’ or antisense 5’-AATTCAAAAAAAGGCAGAGGCTGCCATCTATCTCTTGAATAGATGGCAGCCTCTGCCT-3’).

Exosomes isolation from serum

Six patients (resistant group: n=3; sensitive group: n=3) enrolled in this study were recruited by the Shanghai Chest Hospital. The Ethics committee of Shanghai University of Medicine and Health Sciences approved the study. All patients gave their consent for the collection of blood serum for this study. Chemotherapy resistance or sensitivity was defined as the presence or absence of tumor relapse progression within 6 months,

or 6 months after completion of prior cisplatin-based chemotherapy. 5mL peripheral blood from the patients was collected in anticoagulant tube with EDTA and then was centrifuge at 3000 × g for 10 min to remove debris and cells. Next, the supernatant was mixed with equal volume of PBS and transferred to 50 mL centrifuge tubes for centrifuging 30 min at 2000 × g. Then, the supernatant was centrifuged at 12,000 × g for 45 min followed by ultracentrifugation for 120 min at 110,000 ×g. The pellets were resuspended in a large volume of PBS and then filetered through a 0.22-μm filter. Subsequently, pelleted exosomes were washed twice with PBS and centrifuged at 110,000 ×g for 70 min, then the pelleted exosomes were resuspended in 100 μl PBS and stored at -80 ℃.

Immunoblotting

Cells were lysed using radioimmunoprecipitation assay (RIPA) buffer (KGP704, Keygenbio, Nanjing, China) containing protease inhibitor cocktail (P8340, Sigma-Aldrich). Total protein was quantified using Pierce BCA protein assay kit (Thermo Fisher Scientific) and resolved through an SDS polyacrylamide gel before being transferred onto a PVDF membrane (Millipore, USA). Later, blots were blocked in 5% bovine serum albumin (BSA) for one hour at room temperature and stained with primary antibodies at 4°C overnight, followed by incubation with a secondary antibody for one hour. Images were detected using the bioanalytical imaging system (Azure Biosystems C300). Primary antibodies used are as follows: anti-PKM2 (#4053, Cell Signaling Technology, MA, USA), anti-β-actin (#4970, Cell Signaling Technology, MA, USA), anti-LDHA (#3582, Cell Signaling Technology, MA, USA), anti-HK2 (22029-1-AP,Proteintech, Wuhan, China), anti-GADPH (#5174, Cell Signaling Technology, MA, USA), anti-cleaved caspase 3 (#9579, Cell Signaling Technology, MA, USA), anti-HSP90 (AC88, Abcam, MA, USA), anti-BCL2 p-T69 (Thermo, MA,

USA), anti-Flag (LF304, EpiZyme, Shanghai, China), anti-CD63 (25682-1-AP, Proteintech, Wuhan, China), anti-TSG101 (A1692, ABclonal, Wuhan, China), anti-Caspase 3 (AF1213, Beyotime, Shanghai, China), anti-BAX (AF0054, Beyotime, Shanghai, China) and anti-BCL2 (#15071, Cell Signaling Technology, MA, USA).

Glutaraldehyde cross-linking

0.025% glutaraldehyde to every 4 μg / μl exosomes was added into the lysates and incubated at 37°C for 3min. Then the reaction was stopped with 10μl 1M-Tris-HCl (pH 8.0) and dimeric and tetrameric PKM2 was detected by western blotting.

Quantitative real-time PCR (qRT-PCR)

Total RNA was isolated from control or treated cells using the RNeasy Mini kit (Qiagen, Hilden, Germany). Then, complementary DNA (cDNA) was synthesized using HiScript II Q RT SuperMix (R223-01, Vazyme, Nanjing, China). Real-time PCR was completed using SYBR Green PCR Master Mix (Takara, Otsu, Japan) on QuantStudioTM 7 Flex (Thermo Fisher Scientific). All target genes were normalized to cyclophilin B. Gene expression was quantified using the delta Ct method. Primer sequences were as follows:

PDK1 forward, 5′-GAGAGCCACTATGGAACACCA-3′ and reverse, 5′-GGAGGTCTCAACACGAGGT-3′.

PKM2 forward, 5′-TCGCATGCAGCACCTGATT-3′and reverse, 5′-CCTCGAATAGCTGCAAGTGGTA-3′.

GLUT1 forward, 5′-CTCCGGTATCGTCAACACGG-3′ and reverse, 5′-AAGCCAAAGATGGCCACGAT-3′.

LDHA forward, 5′-GTCAGCATAGCTGTTCCACTTA-3′ and reverse, 5′-AGCTGATCCTTTAGAGTTGCCA-3′.

CCND1 forward, 5′-GCGAGGAACAGAAGTGC-3′ and reverse, 5′-GAGTTGTCGGTGTAGATGC-3′.

MYC forward, 5′-ACACCCTTCTCCCTTCG-3′ and reverse, 5′-CCGCTCCACATACAGTCC-3′.

Cyclophilin B forward, 5′-AGATGTAGGCCGGGTGATCT-3′ and reverse, 5′-CCGCCCTGGATCATGAAGTC-3′.

Transmission electron microscopy

Isolated exosomes resuspended in PBS were placed on copper grids (Zhongjingkeyi Technology, Beijing, China). Then, the copper grids were washed with PBS droplets, following being fixed with 50 μL 1% glutaraldehyde for 5 minutes and washed eight times using ddH_2O. Later, copper grids were placed on 50 μL uranium acetate (pH=7) for 5 minutes and 50 μL methyl cellulose for 10 minutes. Excess liquid on the copper grids was absorbed using a filter paper and they were air dried for 5 minutes. Sample images were observed using transmission electron microscopy (FEI Tecnai G2 Spirit TEM, USA) at 80 kV.

Nanoparticle tracking analysis

After isolating exosomes, the size distribution and concentration were measured using nanoparticle tracking analysis (NTA) with ZetaView PMX 110 (Particle Metrix, Meerbusch, Germany). Samples were diluted using PBS buffer (Biological Industries, Israel), and temperature was maintained around 23°C and 30°C. Data analysis was performed using ZetaView 8.04.02 software.

Co-immunoprecipitation

Exosome treated cells were lysed using RIPA buffer. Cell extracts were immunoprecipitated with anti-PKM2 antibody for 24 hours and then incubated with

protein A/G (Pierce; Thermo Fisher Scientific, Waltham, MA, USA) for 3 hours. The expression levels of BCL2 or Hsp90 were analyzed using immunoblotting.

Cell invasion

Transwell chambers (Corning, New York, NY, USA) were used to perform cell invasion assays. Cells resuspended in 600 μl of DMEM were added to upper chambers and culture medium (containing 90% DMEM and 10% FBS) was added to bottom chambers. Then, cotton swabs were used to removed nonmigrating cells after 24 hours. Next, cells that migrated to the bottom chambers were stained with crystal violet and then fixed with 4% paraformaldehyde for 10 minutes.

Xenograft tumor model

To determine the role of hypoxic A549/CR-derived exosomes in promoting drug resistance *in vivo*, forty 4-week-old, female nude mice (Shanghai Laboratory Animal Center) were randomly selected and subcutaneously inoculated with 5×10^6 A549 cells. After 19 days, 24 mice with similar tumor sizes were randomly selected for drug injection three times a week. A total of 4 mg/kg cisplatin (or vehicle control) was administered via the tail vein and 10 μg of exosomes (or PBS control) were injected around the subcutaneous tumor for each mouse. In addition, 0.1% dimethyl sulfoxide in isotonic saline or PBS was administered intravenously as a vehicle control. Agents were administered every 3 days. After 21 days of treatment, mice were sacrificed and tumors were subjected to histological analyses.

To verify the therapeutic effects of PKM2-IN and cisplatin on cisplatin-resistant tumors, A549/CR cells were stably infected with a lentivirus expressing pLenti-CBh-luc-tCMV-tdTomato-puro vector. A total of 5×10^6 A549/CR cells expressing luciferase were subcutaneously injected into 30 female nude mice. After 20 days, 20 mice with similar tumor sizes were randomly selected and injected with cisplatin (4 mg/kg),

PKM2-IN (2 mg/kg) or a combination of cisplatin and PKM2-IN by intravenous injection three times a week. As a control vehicle, 0.1% dimethyl sulfoxide in isotonic saline was administered intravenously. Approximately, 20 days after administration, tumors were quantified by *in vivo* bioluminescence imaging using the IVIS Spectrum imaging system (PerkinElmer). The sizes and weights of the tumors were measured and were expressed as mean ± standard deviation (S.D.). All animal experiments were completed based on guidelines provided by the Animal Ethics Committee of Shanghai University of Medicine and Health Sciences.

Histological analyses

Tumor tissues resected from nude mice were fixed with 10% formalin solution and embedded in paraffin. Hematoxylin and eosin (HE) staining was used to observe tissue morphology. For immunohistochemistry (IHC), tissue sections were deparaffinized, rehydrated and treated with 3% H_2O_2 for 10 minutes, followed by being treated with 5% BSA for 20 min Then, sections were incubated with antibodies against PKM2, GLUT1 or cleaved-caspase 3 at 4°C overnight. Following incubation with HRP-conjugated secondary antibodies at room temperature, positive signals were visualized using diaminobenzidine chromogen. A TdT-mediated dUTP nick-end labeling (TUNEL) assay was used to assess apoptosis using the In-Situ Cell Death Detection Kit (11684817910, Roche, Switzerland), based on instructions provided by the manufacturer. Tissue sections were dewaxed, hydrated and incubated in proteinase K solution for 20 min at room temperature. TUNEL reaction mixture was added and incubated at room temperature in the dark for 1 h. Converter-pod solution was added and incubated at room temperature for 30 min. Next, DAB staining, hematoxylin re-staining, gradient alcohol dehydration and neutral gum sealing were performed. All

tissue sections were imaged using an Axiophot light microscope (Zeiss, Oberkochen, Germany).

Supplemental Figures

Supplemental Figure S1

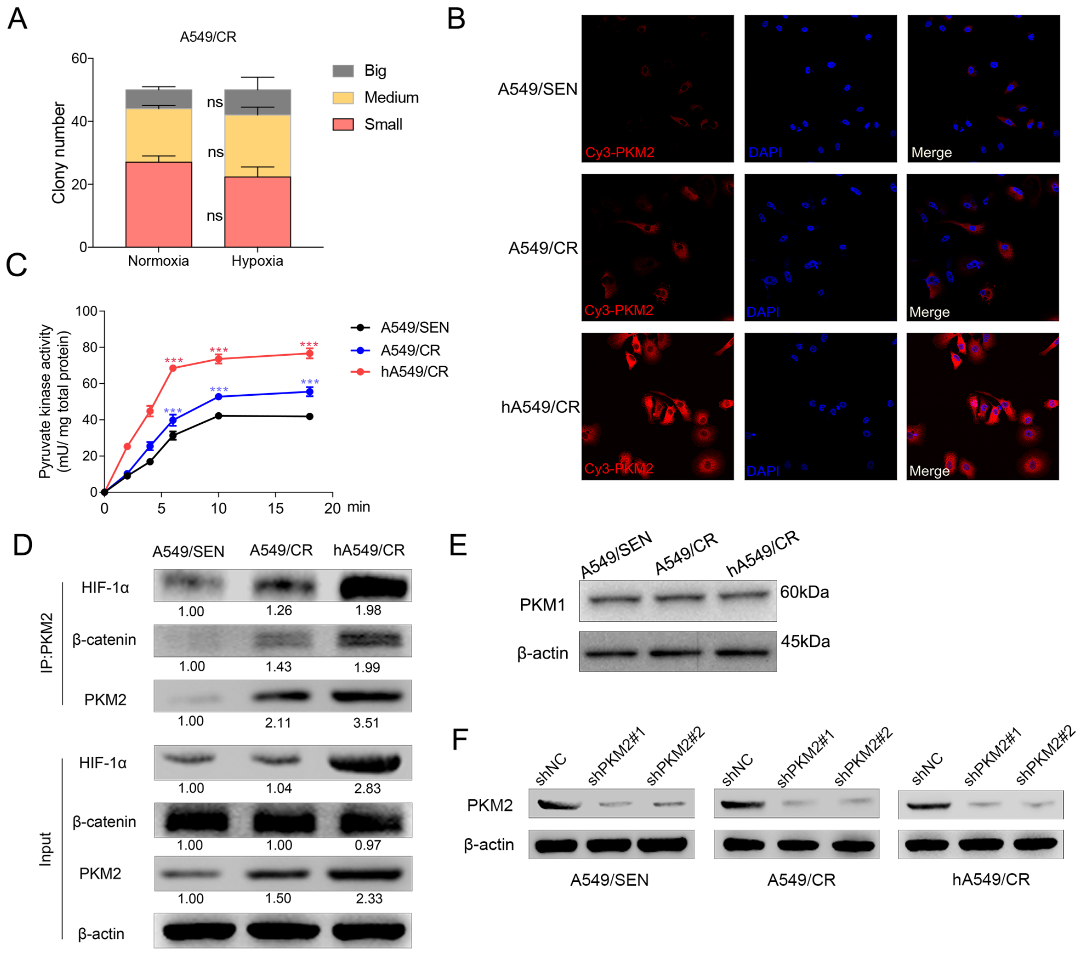

Figure S1. Hypoxia enhances the expression of PKM2 in A549/CR cells. (A) Colony size of A549/CR cells under hypoxia and normoxia conditions. **(B)** Fluorescence confocal microscopy of Cy3-PKM2 (red) in A549/SEN, A549/CR and hA549/CR cells. Cell nuclei were stained with DAPI (blue). **(C)** The activity of PKM2 in A549/SEN, A549/CR and hA549/CR cells. **(D)** PKM2 proteins in A549/SEN, A549/CR and hA549/CR cells were immunoprecipitated and the interacting proteins were detected using immunoblotting. **(E)** Immunoblotting for PKM1 in A549/SEN, A549/CR and hA549/CR cells. **(F)** Immunoblotting for PKM2 in in A549/SEN, A549/CR and hA549/CR cells treated with shNC lentivirus or two different shPKM2 lentiviruses. Data are shown as mean with SD of three independent

experiments. ***p < 0.001; ns, no significance.

Supplemental Figure S2

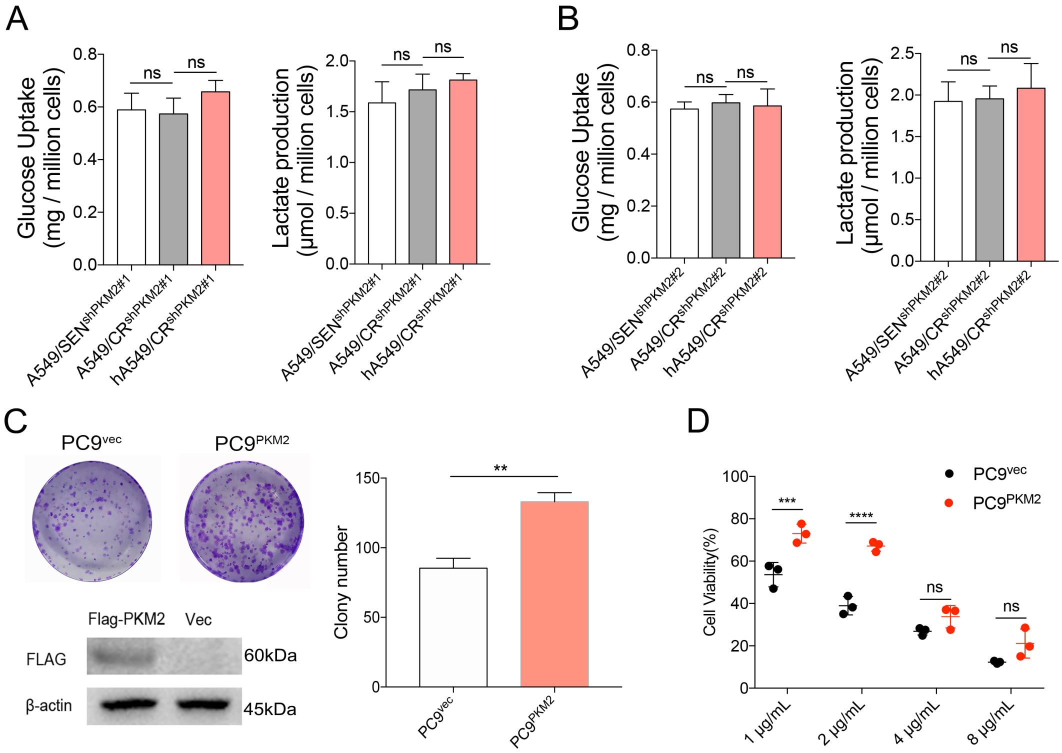

Figure S2. PKM2 promotes the cisplatin-resistance in NSCLC cells. (A-B) A549/SEN, A549/CR and hA549/CR cells treated with shPKM2#1 (A) and shPKM2#2 (B) lentiviruses were cultured for 24 h and then serum-free medium were used for glucose uptake assays and lactate production assays. **(C)** Relative colony numbers and representative images of PC9 cells transfected with vector ($PC9^{vec}$) or Flag-PKM2 ($PC9^{PKM2}$) treated with 2 μg/mL cisplatin. **(D)** Cell viability of $PC9^{vec}$ and $PC9^{PKM2}$ cells treated with different concentrations of cisplatin for 48 h under hypoxic conditions. Data are shown as mean with SD of three independent experiments. **p < 0.01; ***p < 0.001; ****p < 0.0001; ns, no significance.

Supplemental Figure S3

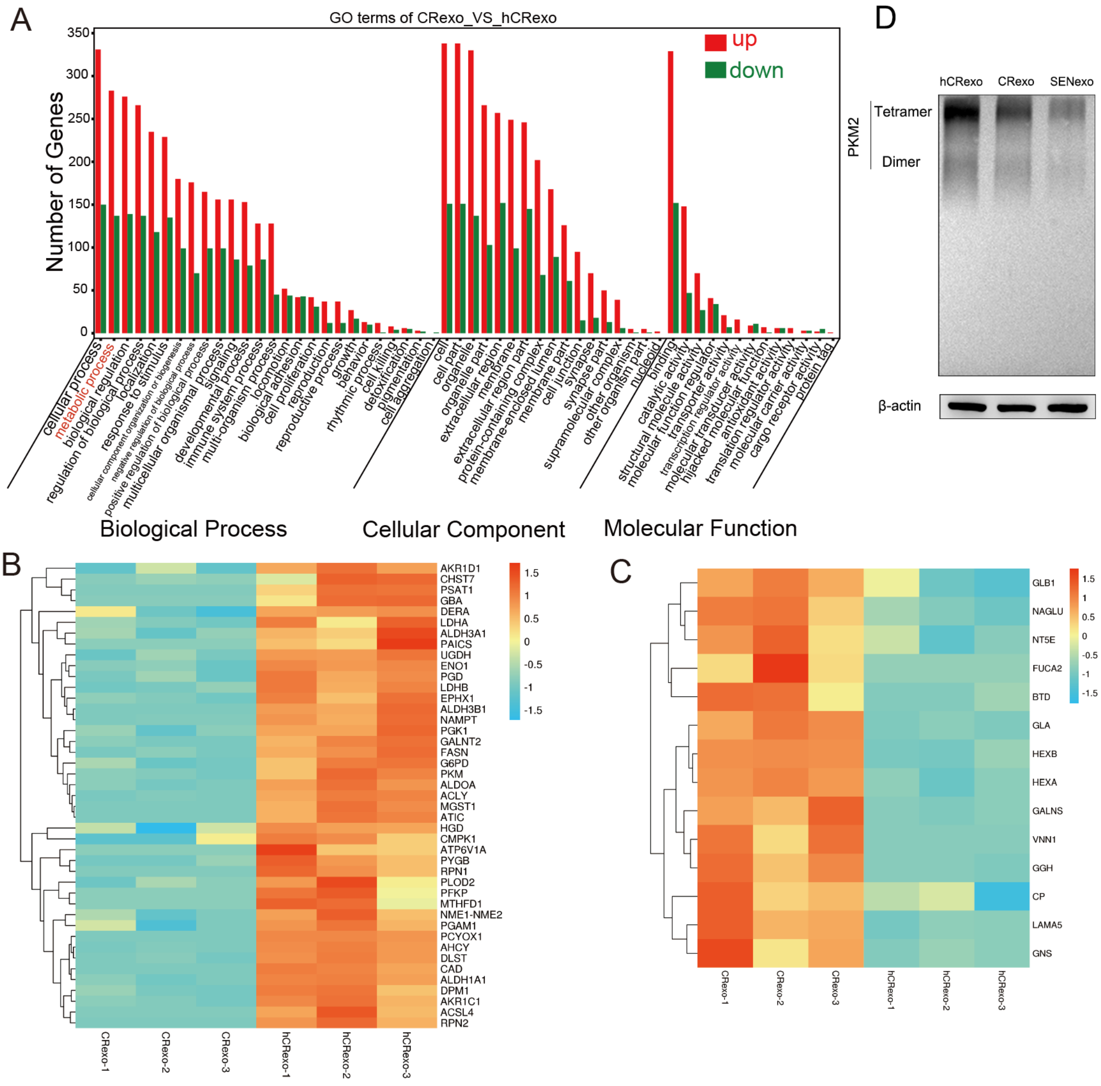

Figure S3. Characterization of exosomal proteins. (A) GO functional classification of differential proteins in CRexo and hCRexo. **(B-C)** A heat map reflected significantly high-expressed proteins in hCRexo (B) and high-expressed proteins in CRexo (C) involved in glucose metabolism. **(D)** The molecular size of PKM2 in SENexo, CRexo and hCRexo was detected by western blotting. Data are shown as mean with SD of three independent experiments.

Supplemental Figure S4

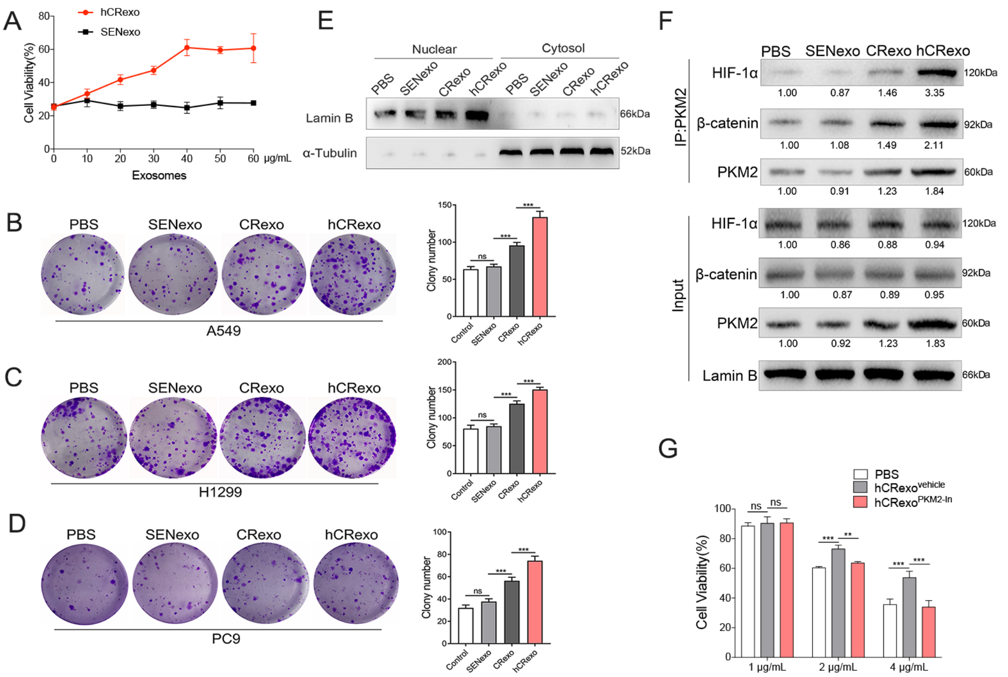

Figure S4. hCRexo promotes the cell proliferation of NSCLC cells under cisplatin treatment. (A) Cell viability of A549/SEN cells treated with different concentrations of exosomes for 48 h under 4 μg/mL cisplatin. **(B-D)** Relative colony numbers (right) and representative images (left) of A549 (A), H1299 (B), PC9 (C) cells treated with PBS, SENexo, CRexo or hCRexo. **(E)** Immunoblotting for α-tubulin and Lamin B in cytoplasmic and nuclear proteins of A549/SEN cells treated with PBS, SENexo, CRexo and hCRexo. **(F)** A549/SEN cells cocultured with 40 μg/mL SENexo, CRexo or hCRexo. PKM2 proteins in nuclear proteins were immunoprecipitated and the interacting proteins were detected using immunoblotting. **(G)** CCK8 assay for A549/SEN cells cocultured with exosomes isolated from hA549/CR cells treated with PKM2 inhibitor or vehicle. Data are shown as mean with SD of three independent experiments. **p <0.01; ***p < 0.001; ns, no significance.

Supplemental Figure S5

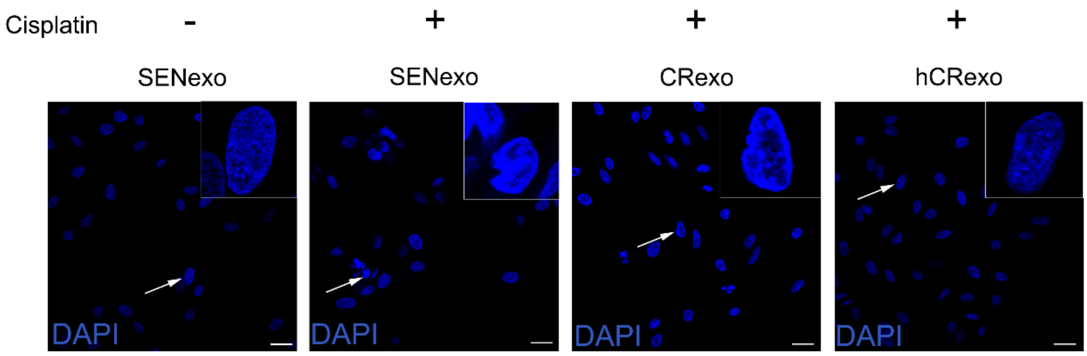

Figure S5. hCRexo inhibits apoptosis induced by cisplatin. Fluorescence confocal microscopy of A549/SEN cells treated with 40 μg/mL SENexo, CRexo and hCRexo under 4 μg/mL cisplatin. Cell nuclei were stained with DAPI (blue).

Supplemental Figure S6

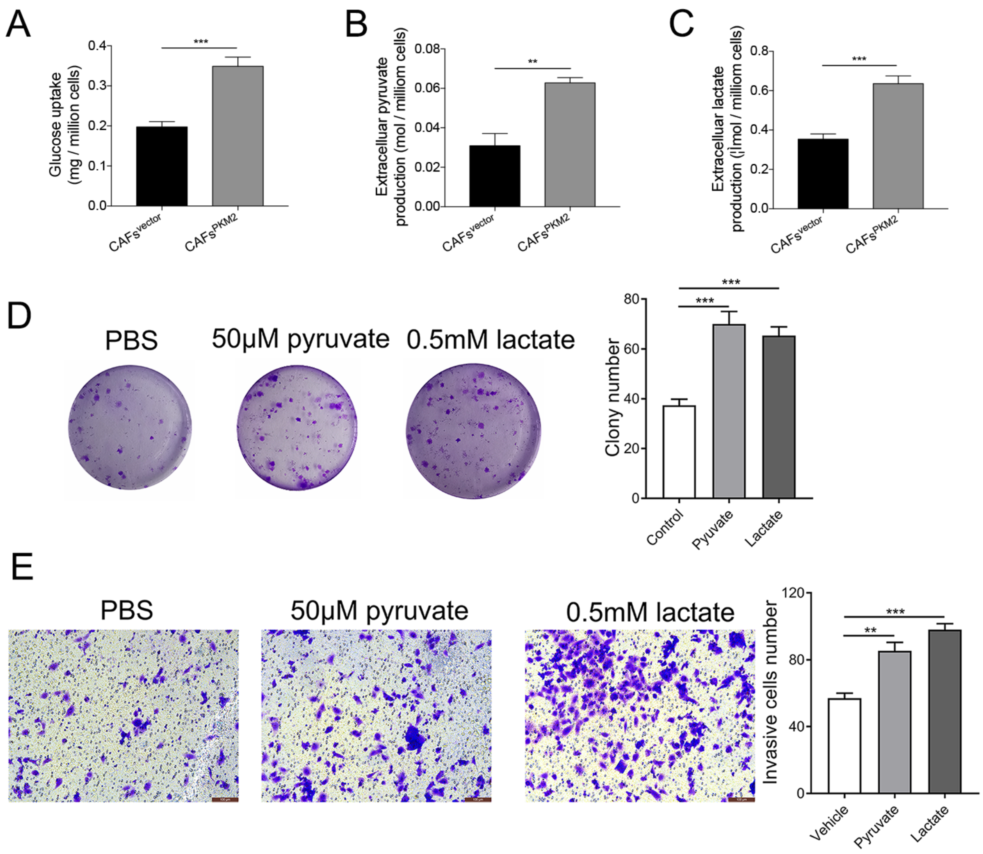

Figure S6. Glycolysis products promotes cell proliferation and invasion of

NSCLC cells. (A-C) The glucose uptake (A), extracellular pyruvate (B) and lactate (C) production in CAFs treated with FLAG-tagged PKM2 or vector. **(D)** Relative colony numbers (right) and representative images (left) of the A549/SEN cells treated with PBS, 50 μM pyruvate or 0.5 mM lactate. **(E)** Transwell assay images (left) and invasive cells number (right) of the above-treated cells. Data are shown as mean with SD of three independent experiments. $^{**}p < 0.01$; $^{***}p < 0.001$.

Supplemental Figure S7

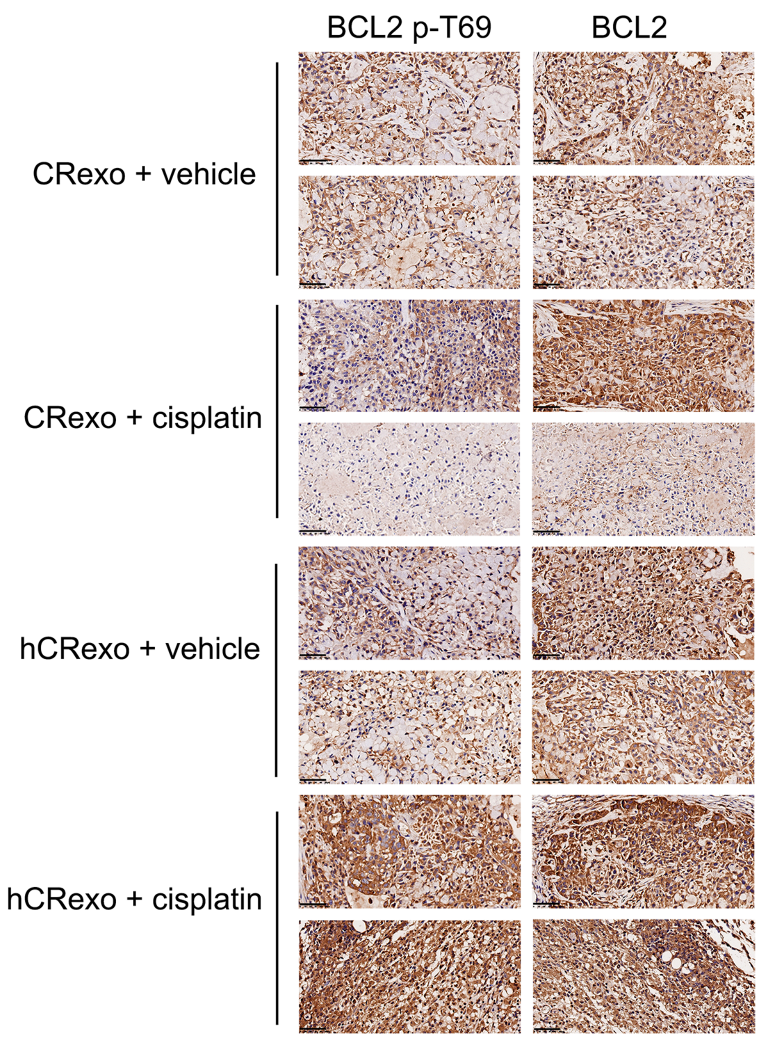

Figure S7. hCRexo significantly increased BCL2 and phosphorylated BCL2

levels in tumor tissues. IHC assays for BCL2 and BCL2 p-T69 detection of tumor tissues. Scale bar, 50 μm.

Supplementary Tables

Table S1. 339 highly expressed proteins in hCRexo identified by LC-MS/MS

No.	Symbol	Description	Average iBAQ of CRexo	Average iBA of hCRexo
1	DPM1	dolichyl-phosphate mannosyltransferase subunit 1, catalytic [Source:HGNC Symbol;Acc:HGNC:3005]	4914533	40375000
2	M6PR	mannose-6-phosphate receptor, cation dependent [Source:HGNC Symbol;Acc:HGNC:6752]	1570100	5393833
3	ITGA3	integrin subunit alpha 3 [Source:HGNC Symbol;Acc:HGNC:6139]	1853567	5650500
4	LAMP2	lysosomal associated membrane protein 2 [Source:HGNC Symbol;Acc:HGNC:6501]	957977	3455033
5	ALDH3B1	aldehyde dehydrogenase 3 family member B1 [Source:HGNC Symbol;Acc:HGNC:410]	0	700003
6	MGST1	microsomal glutathione S-transferase 1 [Source:HGNC Symbol;Acc:HGNC:7061]	0	10488600
7	RPS20	ribosomal protein S20 [Source:HGNC Symbol;Acc:HGNC:10405]	0	24742333
8	NME1-NME2	NME1-NME2 readthrough [Source:HGNC Symbol;Acc:HGNC:33531]	7271700	15688667
9	MVP	major vault protein [Source:HGNC Symbol;Acc:HGNC:7531]	15834000	66618333
10	GPRC5A	G protein-coupled receptor class C group 5 member A [Source:HGNC Symbol;Acc:HGNC:9836]	15496333	24697667
11	ERP44	endoplasmic reticulum protein 44 [Source:HGNC Symbol;Acc:HGNC:18311]	0	2003767
12	DERA	deoxyribose-phosphate aldolase [Source:HGNC Symbol;Acc:HGNC:24269]	534885	1222667
13	EHD2	EH domain containing 2 [Source:HGNC Symbol;Acc:HGNC:3243]	0	628277
14	VIM	vimentin [Source:HGNC Symbol;Acc:HGNC:12692]	208760	1810867
15	VCL	vinculin [Source:HGNC Symbol;Acc:HGNC:12665]	197788	368443
16	C6	complement C6 [Source:HGNC Symbol;Acc:HGNC:1339]	0	97755
17	PSMA4	proteasome subunit alpha 4 [Source:HGNC Symbol;Acc:HGNC:9533]	27433333	39015333
18	HSPA5	heat shock protein family A (Hsp70) member 5 [Source:HGNC Symbol;Acc:HGNC:5238]	5420200	12691000
19	RCN1	reticulocalbin 1 [Source:HGNC Symbol;Acc:HGNC:9934]	0	2559133
20	PHPT1	phosphohistidine phosphatase 1 [Source:HGNC Symbol;Acc:HGNC:30033]	1195567	2433233

21	SLC2A3	solute carrier family 2 member 3 [Source:HGNC Symbol;Acc:HGNC:11007]	0	1800100
22	RPL18	ribosomal protein L18 [Source:HGNC Symbol;Acc:HGNC:10310]	800460	56331333
23	IPO5	importin 5 [Source:HGNC Symbol;Acc:HGNC:6402]	0	70015
24	KARS	lysyl-tRNA synthetase [Source:HGNC Symbol;Acc:HGNC:6215]	0	280803
25	YBX1	Y-box binding protein 1 [Source:HGNC Symbol;Acc:HGNC:8014]	0	1513030
26	PFKP	phosphofructokinase, platelet [Source:HGNC Symbol;Acc:HGNC:8878]	0	1369820
27	PKM	pyruvate kinase M1/2 [Source:HGNC Symbol;Acc:HGNC:9021]	10176067	33765000
28	RHOA	ras homolog family member A [Source:HGNC Symbol;Acc:HGNC:667]	7234033	12328700
29	ACSL4	acyl-CoA synthetase long chain family member 4 [Source:HGNC Symbol;Acc:HGNC:3571]	0	169567
30	ATP1B3	ATPase Na+/K+ transporting subunit beta 3 [Source:HGNC Symbol;Acc:HGNC:806]	0	1114317
31	PABPC1	poly(A) binding protein cytoplasmic 1 [Source:HGNC Symbol;Acc:HGNC:8554]	0	505553
32	CDC42	cell division cycle 42 [Source:HGNC Symbol;Acc:HGNC:1736]	804055	4123967
33	ACTN1	actinin alpha 1 [Source:HGNC Symbol;Acc:HGNC:163]	401930	2096133
34	ENO1	enolase 1 [Source:HGNC Symbol;Acc:HGNC:3350]	12219333	45214000
35	ACTB	actin beta [Source:HGNC Symbol;Acc:HGNC:132]	100171333	200603333
36	XRCC5	X-ray repair cross complementing 5 [Source:HGNC Symbol;Acc:HGNC:12833]	0	267753
37	CLDND1	claudin domain containing 1 [Source:HGNC Symbol;Acc:HGNC:1322]	553840	811960
38	HSP90AA1	heat shock protein 90 alpha family class A member 1 [Source:HGNC Symbol;Acc:HGNC:5253]	0	1684767
39	GSK3B	glycogen synthase kinase 3 beta [Source:HGNC Symbol;Acc:HGNC:4617]	575260	1112423
40	RPS5	ribosomal protein S5 [Source:HGNC Symbol;Acc:HGNC:10426]	0	23392667
41	RAB10	RAB10, member RAS oncogene family [Source:HGNC Symbol;Acc:HGNC:9759]	1029413	13808667
42	CAD	carbamoyl-phosphate synthetase 2, aspartate transcarbamylase, and dihydroorotase [Source:HGNC Symbol;Acc:HGNC:1424]	0	50103
43	EPS15	epidermal growth factor receptor pathway substrate 15 [Source:HGNC Symbol;Acc:HGNC:3419]	0	1278667
44	TMED2	transmembrane p24 trafficking protein 2 [Source:HGNC Symbol;Acc:HGNC:16996]	0	4654667

45	PSMC5	proteasome 26S subunit, ATPase 5 [Source:HGNC Symbol;Acc:HGNC:9552]	0	649667
46	GNAS	GNAS complex locus [Source:HGNC Symbol;Acc:HGNC:4392]	537025	1847533
47	RPL6	ribosomal protein L6 [Source:HGNC Symbol;Acc:HGNC:10362]	0	15980000
48	RPLP0	ribosomal protein lateral stalk subunit P0 [Source:HGNC Symbol;Acc:HGNC:10371]	1253533	51759333
49	ITGA6	integrin subunit alpha 6 [Source:HGNC Symbol;Acc:HGNC:6142]	934060	2517133
50	MYL6	myosin light chain 6 [Source:HGNC Symbol;Acc:HGNC:7587]	489470	1051377
51	DPYSL2	dihydropyrimidinase like 2 [Source:HGNC Symbol;Acc:HGNC:3014]	0	1850267
52	ARCN1	archain 1 [Source:HGNC Symbol;Acc:HGNC:649]	0	281077
53	HSP90AB1	heat shock protein 90 alpha family class B member 1 [Source:HGNC Symbol;Acc:HGNC:5258]	469233	10935967
54	TSPAN15	tetraspanin 15 [Source:HGNC Symbol;Acc:HGNC:23298]	312580	1095520
55	PSMD8	proteasome 26S subunit, non-ATPase 8 [Source:HGNC Symbol;Acc:HGNC:9566]	0	350433
56	EIF3L	eukaryotic translation initiation factor 3 subunit L [Source:HGNC Symbol;Acc:HGNC:18138]	0	704560
57	PACSIN2	protein kinase C and casein kinase substrate in neurons 2 [Source:HGNC Symbol;Acc:HGNC:8571]	487070	1791233
58	RPL3	ribosomal protein L3 [Source:HGNC Symbol;Acc:HGNC:10332]	0	7114000
59	MYH9	myosin heavy chain 9 [Source:HGNC Symbol;Acc:HGNC:7579]	335845	1064443
60	PRMT5	protein arginine methyltransferase 5 [Source:HGNC Symbol;Acc:HGNC:10894]	0	492200
61	PSMC6	proteasome 26S subunit, ATPase 6 [Source:HGNC Symbol;Acc:HGNC:9553]	105860	636733
62	MTHFD1	methylenetetrahydrofolate dehydrogenase, cyclohydrolase and formyltetrahydrofolate synthetase 1 [Source:HGNC Symbol;Acc:HGNC:7432]	0	347153
63	PSMB5	proteasome subunit beta 5 [Source:HGNC Symbol;Acc:HGNC:9542]	12937333	39480000
64	PYGB	glycogen phosphorylase B [Source:HGNC Symbol;Acc:HGNC:9723]	247550	1695733
65	PSMA7	proteasome subunit alpha 7 [Source:HGNC Symbol;Acc:HGNC:9536]	38121667	79496000
66	AHCY	adenosylhomocysteinase [Source:HGNC Symbol;Acc:HGNC:343]	2883167	8924067

67	F9	coagulation factor IX [Source:HGNC Symbol;Acc:HGNC:3551]	0	485643
68	PLP2	proteolipid protein 2 [Source:HGNC Symbol;Acc:HGNC:9087]	0	15061000
69	PGK1	phosphoglycerate kinase 1 [Source:HGNC Symbol;Acc:HGNC:8896]	4004267	9098200
70	PARP4	poly(ADP-ribose) polymerase family member 4 [Source:HGNC Symbol;Acc:HGNC:271]	0	173113
71	ABCC1	ATP binding cassette subfamily C member 1 [Source:HGNC Symbol;Acc:HGNC:51]	0	216070
72	SLC7A5	solute carrier family 7 member 5 [Source:HGNC Symbol;Acc:HGNC:11063]	4376667	12200333
73	CA2	carbonic anhydrase 2 [Source:HGNC Symbol;Acc:HGNC:1373]	0	880820
74	RPS16	ribosomal protein S16 [Source:HGNC Symbol;Acc:HGNC:10396]	1065527	20531667
75	SLC1A5	solute carrier family 1 member 5 [Source:HGNC Symbol;Acc:HGNC:10943]	2335433	11915667
76	RPS19	ribosomal protein S19 [Source:HGNC Symbol;Acc:HGNC:10402]	0	9441800
77	RPL18A	ribosomal protein L18a [Source:HGNC Symbol;Acc:HGNC:10311]	0	3007533
78	NAMPT	nicotinamide phosphoribosyltransferase [Source:HGNC Symbol;Acc:HGNC:30092]	195077	2029300
79	CAV1	caveolin 1 [Source:HGNC Symbol;Acc:HGNC:1527]	1033900	25042000
80	DNAJB6	DnaJ heat shock protein family (Hsp40) member B6 [Source:HGNC Symbol;Acc:HGNC:14888]	0	756940
81	SSBP1	single stranded DNA binding protein 1 [Source:HGNC Symbol;Acc:HGNC:11317]	0	7107000
82	HSPB1	heat shock protein family B (small) member 1 [Source:HGNC Symbol;Acc:HGNC:5246]	0	2524900
83	EIF3B	eukaryotic translation initiation factor 3 subunit B [Source:HGNC Symbol;Acc:HGNC:3280]	0	531143
84	AGR2	anterior gradient 2, protein disulphide isomerase family member [Source:HGNC Symbol;Acc:HGNC:328]	3867900	8105600
85	PTGR1	prostaglandin reductase 1 [Source:HGNC Symbol;Acc:HGNC:18429]	601270	1498100
86	EIF3A	eukaryotic translation initiation factor 3 subunit A [Source:HGNC Symbol;Acc:HGNC:3271]	0	228403
87	TSPAN14	tetraspanin 14 [Source:HGNC Symbol;Acc:HGNC:23303]	3486390	8483600
88	RPL19	ribosomal protein L19 [Source:HGNC Symbol;Acc:HGNC:10312]	0	25988333
89	PSMD3	proteasome 26S subunit, non-ATPase 3 [Source:HGNC Symbol;Acc:HGNC:9560]	0	1067370

90	KPNB1	karyopherin subunit beta 1 [Source:HGNC Symbol;Acc:HGNC:6400]	104029	886223
91	PFN1	profilin 1 [Source:HGNC Symbol;Acc:HGNC:8881]	2941033	4838800
92	C1QBP	complement C1q binding protein [Source:HGNC Symbol;Acc:HGNC:1243]	0	2308233
93	ALDH3A1	aldehyde dehydrogenase 3 family member A1 [Source:HGNC Symbol;Acc:HGNC:405]	16079000	24854000
94	SYNGR2	synaptogyrin 2 [Source:HGNC Symbol;Acc:HGNC:11499]	743900	1500300
95	RAB5C	RAB5C, member RAS oncogene family [Source:HGNC Symbol;Acc:HGNC:9785]	2030167	4036600
96	VAT1	vesicle amine transport 1 [Source:HGNC Symbol;Acc:HGNC:16919]	366200	1453100
97	EFTUD2	elongation factor Tu GTP binding domain containing 2 [Source:HGNC Symbol;Acc:HGNC:30858]	0	321353
98	RPL34	ribosomal protein L34 [Source:HGNC Symbol;Acc:HGNC:10340]	0	12544333
99	DHX15	DEAH-box helicase 15 [Source:HGNC Symbol;Acc:HGNC:2738]	493917	2205267
100	UGDH	UDP-glucose 6-dehydrogenase [Source:HGNC Symbol;Acc:HGNC:12525]	1595770	9494000
101	EHD1	EH domain containing 1 [Source:HGNC Symbol;Acc:HGNC:3242]	709797	1616433
102	RPS13	ribosomal protein S13 [Source:HGNC Symbol;Acc:HGNC:10386]	0	13085500
103	KRT18	keratin 18 [Source:HGNC Symbol;Acc:HGNC:6430]	3842300	19379333
104	GOLT1B	golgi transport 1B [Source:HGNC Symbol;Acc:HGNC:20175]	0	1533567
105	LDHB	lactate dehydrogenase B [Source:HGNC Symbol;Acc:HGNC:6541]	16248333	63042333
106	SERINC1	serine incorporator 1 [Source:HGNC Symbol;Acc:HGNC:13464]	0	571457
107	SRSF3	serine and arginine rich splicing factor 3 [Source:HGNC Symbol;Acc:HGNC:10785]	0	6786233
108	SUB1	SUB1 regulator of transcription [Source:HGNC Symbol;Acc:HGNC:19985]	0	3873833
109	TARS	threonyl-tRNA synthetase [Source:HGNC Symbol;Acc:HGNC:11572]	98018	394247
110	C9	complement C9 [Source:HGNC Symbol;Acc:HGNC:1358]	0	239240
111	RARS	arginyl-tRNA synthetase [Source:HGNC Symbol;Acc:HGNC:9870]	0	518640
112	HGD	homogentisate 1,2-dioxygenase [Source:HGNC Symbol;Acc:HGNC:4892]	509515	905583
113	GNAI2	G protein subunit alpha i2 [Source:HGNC Symbol;Acc:HGNC:4385]	0	842433

114	ATP6V1A	ATPase H+ transporting V1 subunit A [Source:HGNC Symbol;Acc:HGNC:851]	0	197707
115	MOB1A	MOB kinase activator 1A [Source:HGNC Symbol;Acc:HGNC:16015]	0	510790
116	NCL	nucleolin [Source:HGNC Symbol;Acc:HGNC:7667]	0	5329833
117	LANCL1	LanC like 1 [Source:HGNC Symbol;Acc:HGNC:6508]	0	1302967
118	CCT4	chaperonin containing TCP1 subunit 4 [Source:HGNC Symbol;Acc:HGNC:1617]	243230	862267
119	SF3B1	splicing factor 3b subunit 1 [Source:HGNC Symbol;Acc:HGNC:10768]	0	136780
120	DARS	aspartyl-tRNA synthetase [Source:HGNC Symbol;Acc:HGNC:2678]	0	1364530
121	PCYOX1	prenylcysteine oxidase 1 [Source:HGNC Symbol;Acc:HGNC:20588]	0	426733
122	QSOX1	quiescin sulfhydryl oxidase 1 [Source:HGNC Symbol;Acc:HGNC:9756]	773130	1364167
123	PARK7	Parkinsonism associated deglycase [Source:HGNC Symbol;Acc:HGNC:16369]	604295	1616367
124	CRYZ	crystallin zeta [Source:HGNC Symbol;Acc:HGNC:2419]	0	486213
125	PRDX1	peroxiredoxin 1 [Source:HGNC Symbol;Acc:HGNC:9352]	4736567	15704000
126	RPS25	ribosomal protein S25 [Source:HGNC Symbol;Acc:HGNC:10413]	0	25988333
127	RPN2	ribophorin II [Source:HGNC Symbol;Acc:HGNC:10382]	0	332137
128	SET	SET nuclear proto-oncogene [Source:HGNC Symbol;Acc:HGNC:10760]	0	8595967
129	DLST	dihydrolipoamide S-succinyltransferase [Source:HGNC Symbol;Acc:HGNC:2911]	401493	2825300
130	TCP1	t-complex 1 [Source:HGNC Symbol;Acc:HGNC:11655]	116076	1447433
131	ANXA11	annexin A11 [Source:HGNC Symbol;Acc:HGNC:535]	0	5929400
132	RPL5	ribosomal protein L5 [Source:HGNC Symbol;Acc:HGNC:10360]	0	20194000
133	HNRNPA2B1	heterogeneous nuclear ribonucleoprotein A2/B1 [Source:HGNC Symbol;Acc:HGNC:5033]	226430	1669133
134	AKR1D1	aldo-keto reductase family 1 member D1 [Source:HGNC Symbol;Acc:HGNC:388]	538070	1285267
135	PRDX4	peroxiredoxin 4 [Source:HGNC Symbol;Acc:HGNC:17169]	994750	4800767
136	ADGRE5	adhesion G protein-coupled receptor E5 [Source:HGNC Symbol;Acc:HGNC:1711]	189590	785360
137	DNPEP	aspartyl aminopeptidase [Source:HGNC Symbol;Acc:HGNC:2981]	601157	1924433
138	SSR1	signal sequence receptor subunit 1 [Source:HGNC Symbol;Acc:HGNC:11323]	0	478737
139	AHNAK	AHNAK nucleoprotein [Source:HGNC Symbol;Acc:HGNC:347]	15189	563430

140	HNRNPR	heterogeneous nuclear ribonucleoprotein R [Source:HGNC Symbol;Acc:HGNC:5047]	0	129867
141	CAPNS1	calpain small subunit 1 [Source:HGNC Symbol;Acc:HGNC:1481]	0	862213
142	RRAS	RAS related [Source:HGNC Symbol;Acc:HGNC:10447]	218095	1285100
143	CANX	calnexin [Source:HGNC Symbol;Acc:HGNC:1473]	166920	5063767
144	MASP1	mannan binding lectin serine peptidase 1 [Source:HGNC Symbol;Acc:HGNC:6901]	222877	701473
145	TUBA4A	tubulin alpha 4a [Source:HGNC Symbol;Acc:HGNC:12407]	548040	3594800
146	PAICS	phosphoribosylaminoimidazole carboxylase and phosphoribosylaminoimidazolesuccinocarboxamide synthase [Source:HGNC Symbol;Acc:HGNC:8587]	0	429983
147	COPB1	coatomer protein complex subunit beta 1 [Source:HGNC Symbol;Acc:HGNC:2231]	0	238183
148	PSMA1	proteasome subunit alpha 1 [Source:HGNC Symbol;Acc:HGNC:9530]	23746667	36533000
149	MYOD1	myogenic differentiation 1 [Source:HGNC Symbol;Acc:HGNC:7611]	12652633	31263667
150	LBP	lipopolysaccharide binding protein [Source:HGNC Symbol;Acc:HGNC:6517]	735163	4290000
151	PRKCSH	protein kinase C substrate 80K-H [Source:HGNC Symbol;Acc:HGNC:9411]	0	858287
152	ACTN4	actinin alpha 4 [Source:HGNC Symbol;Acc:HGNC:166]	13362667	22923000
153	EIF2S3	eukaryotic translation initiation factor 2 subunit gamma [Source:HGNC Symbol;Acc:HGNC:3267]	0	653233
154	UBA1	ubiquitin like modifier activating enzyme 1 [Source:HGNC Symbol;Acc:HGNC:12469]	76903	516917
155	CAP1	cyclase associated actin cytoskeleton regulatory protein 1 [Source:HGNC Symbol;Acc:HGNC:20040]	123180	2324933
156	SLC6A6	solute carrier family 6 member 6 [Source:HGNC Symbol;Acc:HGNC:11052]	0	855807
157	RPL27	ribosomal protein L27 [Source:HGNC Symbol;Acc:HGNC:10328]	0	17856333
158	ACLY	ATP citrate lyase [Source:HGNC Symbol;Acc:HGNC:115]	491480	6941967
159	IQCA1	IQ motif containing with AAA domain 1 [Source:HGNC Symbol;Acc:HGNC:26195]	3014967	24408333
160	ITGB4	integrin subunit beta 4 [Source:HGNC Symbol;Acc:HGNC:6158]	0	74312
161	EIF2S1	eukaryotic translation initiation factor 2 subunit alpha [Source:HGNC Symbol;Acc:HGNC:3265]	0	348017
162	YWHAQ	tyrosine 3-monooxygenase/tryptophan 5-monooxygenase activation protein theta [Source:HGNC Symbol;Acc:HGNC:12854]	1029740	5847667

163	LDHA	lactate dehydrogenase A [Source:HGNC Symbol;Acc:HGNC:6535]	24094000	62720000
164	RPS15A	ribosomal protein S15a [Source:HGNC Symbol;Acc:HGNC:10389]	0	19259333
165	TPP2	tripeptidyl peptidase 2 [Source:HGNC Symbol;Acc:HGNC:12016]	0	142447
166	ANXA1	annexin A1 [Source:HGNC Symbol;Acc:HGNC:533]	44980667	351130000
167	PSAT1	phosphoserine aminotransferase 1 [Source:HGNC Symbol;Acc:HGNC:19129]	0	474870
168	TMBIM1	transmembrane BAX inhibitor motif containing 1 [Source:HGNC Symbol;Acc:HGNC:23410]	1159250	2540567
169	FLNB	filamin B [Source:HGNC Symbol;Acc:HGNC:3755]	129820	560883
170	ITM2B	integral membrane protein 2B [Source:HGNC Symbol;Acc:HGNC:6174]	338240	1020703
171	RAC1	Rac family small GTPase 1 [Source:HGNC Symbol;Acc:HGNC:9801]	2064933	7130000
172	TTYH3	tweety family member 3 [Source:HGNC Symbol;Acc:HGNC:22222]	323227	805590
173	PSMB7	proteasome subunit beta 7 [Source:HGNC Symbol;Acc:HGNC:9544]	10808067	49144333
174	ANP32B	acidic nuclear phosphoprotein 32 family member B [Source:HGNC Symbol;Acc:HGNC:16677]	0	2805067
175	RPS6	ribosomal protein S6 [Source:HGNC Symbol;Acc:HGNC:10429]	0	20228333
176	MYOF	myoferlin [Source:HGNC Symbol;Acc:HGNC:3656]	0	2033467
177	ANXA7	annexin A7 [Source:HGNC Symbol;Acc:HGNC:545]	0	2596067
178	ATIC	5-aminoimidazole-4-carboxamide ribonucleotide formyltransferase/IMP cyclohydrolase [Source:HGNC Symbol;Acc:HGNC:794]	0	1620500
179	OLA1	Obg like ATPase 1 [Source:HGNC Symbol;Acc:HGNC:28833]	0	109785
180	SCARB2	scavenger receptor class B member 2 [Source:HGNC Symbol;Acc:HGNC:1665]	286630	1190717
181	ANXA3	annexin A3 [Source:HGNC Symbol;Acc:HGNC:541]	560610	9531133
182	CPNE8	copine 8 [Source:HGNC Symbol;Acc:HGNC:23498]	170950	2667867
183	VPS37B	VPS37B subunit of ESCRT-I [Source:HGNC Symbol;Acc:HGNC:25754]	2208433	4352667
184	ANP32A	acidic nuclear phosphoprotein 32 family member A [Source:HGNC Symbol;Acc:HGNC:13233]	0	1025983
185	ETFA	electron transfer flavoprotein subunit alpha [Source:HGNC Symbol;Acc:HGNC:3481]	0	1751900
186	WDR61	WD repeat domain 61 [Source:HGNC Symbol;Acc:HGNC:30300]	0	4223100

187	IQGAP1	IQ motif containing GTPase activating protein 1 [Source:HGNC Symbol;Acc:HGNC:6110]	0	567700
188	RPS2	ribosomal protein S2 [Source:HGNC Symbol;Acc:HGNC:10404]	0	26606667
189	NPEPPS	aminopeptidase puromycin sensitive [Source:HGNC Symbol;Acc:HGNC:7900]	0	129077
190	CLTC	clathrin heavy chain [Source:HGNC Symbol;Acc:HGNC:2092]	1069627	14874667
191	SOD1	superoxide dismutase 1 [Source:HGNC Symbol;Acc:HGNC:11179]	0	6349133
192	PSMB6	proteasome subunit beta 6 [Source:HGNC Symbol;Acc:HGNC:9543]	11111600	27927667
193	RPS11	ribosomal protein S11 [Source:HGNC Symbol;Acc:HGNC:10384]	0	5552567
194	RPL13A	ribosomal protein L13a [Source:HGNC Symbol;Acc:HGNC:10304]	0	4833633
195	PGD	phosphogluconate dehydrogenase [Source:HGNC Symbol;Acc:HGNC:8891]	5811633	21949667
196	RPS8	ribosomal protein S8 [Source:HGNC Symbol;Acc:HGNC:10441]	0	27484333
197	ATP1B1	ATPase Na+/K+ transporting subunit beta 1 [Source:HGNC Symbol;Acc:HGNC:804]	1331260	5181900
198	HDGF	heparin binding growth factor [Source:HGNC Symbol;Acc:HGNC:4856]	0	383070
199	ACTC1	actin alpha cardiac muscle 1 [Source:HGNC Symbol;Acc:HGNC:143]	8881500	31789000
200	GALNT2	polypeptide N-acetylgalactosaminyltransferase 2 [Source:HGNC Symbol;Acc:HGNC:4124]	158730	2258667
201	EPHX1	epoxide hydrolase 1 [Source:HGNC Symbol;Acc:HGNC:3401]	451970	4393733
202	PDIA6	protein disulfide isomerase family A member 6 [Source:HGNC Symbol;Acc:HGNC:30168]	0	936717
203	RPS27A	ribosomal protein S27a [Source:HGNC Symbol;Acc:HGNC:10417]	419086667	582890000
204	ARL6IP5	ADP ribosylation factor like GTPase 6 interacting protein 5 [Source:HGNC Symbol;Acc:HGNC:16937]	0	4887867
205	CISD2	CDGSH iron sulfur domain 2 [Source:HGNC Symbol;Acc:HGNC:24212]	0	937440
206	RPS3A	ribosomal protein S3A [Source:HGNC Symbol;Acc:HGNC:10421]	0	8701833
207	EGFR	epidermal growth factor receptor [Source:HGNC Symbol;Acc:HGNC:3236]	263034	1520800
208	IGFBP3	insulin like growth factor binding protein 3 [Source:HGNC Symbol;Acc:HGNC:5472]	2700000	8373467

209	IGFBP1	insulin like growth factor binding protein 1 [Source:HGNC Symbol;Acc:HGNC:5469]	4391367	13832333
210	CCT6A	chaperonin containing TCP1 subunit 6A [Source:HGNC Symbol;Acc:HGNC:1620]	575547	2500467
211	CHST7	carbohydrate sulfotransferase 7 [Source:HGNC Symbol;Acc:HGNC:13817]	1917267	3471833
212	NONO	non-POU domain containing octamer binding [Source:HGNC Symbol;Acc:HGNC:7871]	0	523897
213	RPL10	ribosomal protein L10 [Source:HGNC Symbol;Acc:HGNC:10298]	0	12619667
214	RPL7	ribosomal protein L7 [Source:HGNC Symbol;Acc:HGNC:10363]	0	60115333
215	RPL7A	ribosomal protein L7a [Source:HGNC Symbol;Acc:HGNC:10364]	0	19190667
216	RPS3	ribosomal protein S3 [Source:HGNC Symbol;Acc:HGNC:10420]	259485	14722000
217	ALDOA	aldolase, fructose-bisphosphate A [Source:HGNC Symbol;Acc:HGNC:414]	2820100	43925000
218	ITGB1	integrin subunit beta 1 [Source:HGNC Symbol;Acc:HGNC:6153]	7803300	32171333
219	CCT5	chaperonin containing TCP1 subunit 5 [Source:HGNC Symbol;Acc:HGNC:1618]	92754	606967
220	EPS8	epidermal growth factor receptor pathway substrate 8 [Source:HGNC Symbol;Acc:HGNC:3420]	0	400467
221	PLOD2	procollagen-lysine,2-oxoglutarate 5-dioxygenase 2 [Source:HGNC Symbol;Acc:HGNC:9082]	697840	3510933
222	CXADR	CXADR Ig-like cell adhesion molecule [Source:HGNC Symbol;Acc:HGNC:2559]	0	148437
223	PDIA4	protein disulfide isomerase family A member 4 [Source:HGNC Symbol;Acc:HGNC:30167]	0	920877
224	CCT8	chaperonin containing TCP1 subunit 8 [Source:HGNC Symbol;Acc:HGNC:1623]	325533	2518667
225	RPL30	ribosomal protein L30 [Source:HGNC Symbol;Acc:HGNC:10333]	0	1613333
226	CLDN12	claudin 12 [Source:HGNC Symbol;Acc:HGNC:2034]	0	868733
227	MYO1E	myosin IE [Source:HGNC Symbol;Acc:HGNC:7599]	0	195083
228	HIST1H4B	histone cluster 1 H4 family member b [Source:HGNC Symbol;Acc:HGNC:4789]	152276667	204720000
229	TAGLN2	transgelin 2 [Source:HGNC Symbol;Acc:HGNC:11554]	1429393	6211200
230	CALM3	calmodulin 3 [Source:HGNC Symbol;Acc:HGNC:1449]	38637667	67505000
231	G6PD	glucose-6-phosphate dehydrogenase [Source:HGNC Symbol;Acc:HGNC:4057]	2182667	7572867
232	FCN2	ficolin 2 [Source:HGNC Symbol;Acc:HGNC:3624]	1155700	11434667

233	VPS28	VPS28 subunit of ESCRT-I [Source:HGNC Symbol;Acc:HGNC:18178]	2756100	5984267
234	SQSTM1	sequestosome 1 [Source:HGNC Symbol;Acc:HGNC:11280]	0	834303
235	PSMC2	proteasome 26S subunit, ATPase 2 [Source:HGNC Symbol;Acc:HGNC:9548]	123690	1075423
236	AP2M1	adaptor related protein complex 2 subunit mu 1 [Source:HGNC Symbol;Acc:HGNC:564]	0	493380
237	SRSF2	serine and arginine rich splicing factor 2 [Source:HGNC Symbol;Acc:HGNC:10783]	0	5084633
238	ITGA5	integrin subunit alpha 5 [Source:HGNC Symbol;Acc:HGNC:6141]	0	503833
239	EIF4A1	eukaryotic translation initiation factor 4A1 [Source:HGNC Symbol;Acc:HGNC:3282]	198863	1966967
240	CMPK1	cytidine/uridine monophosphate kinase 1 [Source:HGNC Symbol;Acc:HGNC:18170]	147200	227313
241	NCSTN	nicastrin [Source:HGNC Symbol;Acc:HGNC:17091]	174597	522647
242	S100A11	S100 calcium binding protein A11 [Source:HGNC Symbol;Acc:HGNC:10488]	2666300	28544333
243	ATP1A1	ATPase Na+/K+ transporting subunit alpha 1 [Source:HGNC Symbol;Acc:HGNC:799]	0	2347710
244	ARPC2	actin related protein 2/3 complex subunit 2 [Source:HGNC Symbol;Acc:HGNC:705]	0	996947
245	CCT3	chaperonin containing TCP1 subunit 3 [Source:HGNC Symbol;Acc:HGNC:1616]	0	2709833
246	PSMD6	proteasome 26S subunit, non-ATPase 6 [Source:HGNC Symbol;Acc:HGNC:9564]	0	1001490
247	RPN1	ribophorin I [Source:HGNC Symbol;Acc:HGNC:10381]	0	469877
248	PGRMC2	progesterone receptor membrane component 2 [Source:HGNC Symbol;Acc:HGNC:16089]	0	1070957
249	ANXA5	annexin A5 [Source:HGNC Symbol;Acc:HGNC:543]	3978400	37213000
250	RPS14	ribosomal protein S14 [Source:HGNC Symbol;Acc:HGNC:10387]	0	5532267
251	ALDH1A1	aldehyde dehydrogenase 1 family member A1 [Source:HGNC Symbol;Acc:HGNC:402]	69217667	183386667
252	HNRNPK	heterogeneous nuclear ribonucleoprotein K [Source:HGNC Symbol;Acc:HGNC:5044]	258740	1709633
253	VCP	valosin containing protein [Source:HGNC Symbol;Acc:HGNC:12666]	8936300	37865333
254	PSMC3	proteasome 26S subunit, ATPase 3 [Source:HGNC Symbol;Acc:HGNC:9549]	0	587717
255	CCT2	chaperonin containing TCP1 subunit 2 [Source:HGNC Symbol;Acc:HGNC:1615]	522760	1729133
256	RPL27A	ribosomal protein L27a [Source:HGNC Symbol;Acc:HGNC:10329]	0	12874667

257	HSP90B1	heat shock protein 90 beta family member 1 [Source:HGNC Symbol;Acc:HGNC:12028]	0	3644467
258	PDIA3	protein disulfide isomerase family A member 3 [Source:HGNC Symbol;Acc:HGNC:4606]	115594	1337100
259	RPL13	ribosomal protein L13 [Source:HGNC Symbol;Acc:HGNC:10303]	0	11409333
260	TUBA1C	tubulin alpha 1c [Source:HGNC Symbol;Acc:HGNC:20768]	9743400	28502000
261	DDB1	damage specific DNA binding protein 1 [Source:HGNC Symbol;Acc:HGNC:2717]	177905	783943
262	SLC3A2	solute carrier family 3 member 2 [Source:HGNC Symbol;Acc:HGNC:11026]	27007333	39837333
263	RPSA	ribosomal protein SA [Source:HGNC Symbol;Acc:HGNC:6502]	1439060	9826367
264	ARF4	ADP ribosylation factor 4 [Source:HGNC Symbol;Acc:HGNC:655]	5763933	9570133
265	FASN	fatty acid synthase [Source:HGNC Symbol;Acc:HGNC:3594]	289530	3146533
266	BRD3	bromodomain containing 3 [Source:HGNC Symbol;Acc:HGNC:1104]	26130000	58529000
267	YWHAG	tyrosine 3-monooxygenase/tryptophan 5-monooxygenase activation protein gamma [Source:HGNC Symbol;Acc:HGNC:12852]	3052100	4882333
268	PDCD6IP	programmed cell death 6 interacting protein [Source:HGNC Symbol;Acc:HGNC:8766]	5116067	8509000
269	TMED10	transmembrane p24 trafficking protein 10 [Source:HGNC Symbol;Acc:HGNC:16998]	539900	7793333
270	RPS9	ribosomal protein S9 [Source:HGNC Symbol;Acc:HGNC:10442]	1114900	50353333
271	PGAM1	phosphoglycerate mutase 1 [Source:HGNC Symbol;Acc:HGNC:8888]	1990000	3310833
272	BSG	basigin (Ok blood group) [Source:HGNC Symbol;Acc:HGNC:1116]	5875933	13555667
273	HSPA6	heat shock protein family A (Hsp70) member 6 [Source:HGNC Symbol;Acc:HGNC:5239]	5965400	9220267
274	PSMD1	proteasome 26S subunit, non-ATPase 1 [Source:HGNC Symbol;Acc:HGNC:9554]	0	659777
275	MUC13	mucin 13, cell surface associated [Source:HGNC Symbol;Acc:HGNC:7511]	947127	2720400
276	CNP	2',3'-cyclic nucleotide 3' phosphodiesterase [Source:HGNC Symbol;Acc:HGNC:2158]	2760700	11826000
277	ATP2A2	ATPase sarcoplasmic/endoplasmic reticulum Ca2+ transporting 2 [Source:HGNC Symbol;Acc:HGNC:812]	0	217150
278	RPL4	ribosomal protein L4 [Source:HGNC Symbol;Acc:HGNC:10353]	0	7532467

279	RPL15	ribosomal protein L15 [Source:HGNC Symbol;Acc:HGNC:10306]	0	19436333
280	RAB1B	RAB1B, member RAS oncogene family [Source:HGNC Symbol;Acc:HGNC:18370]	863993	3837967
281	PSMD2	proteasome 26S subunit, non-ATPase 2 [Source:HGNC Symbol;Acc:HGNC:9559]	0	610903
282	RUVBL1	RuvB like AAA ATPase 1 [Source:HGNC Symbol;Acc:HGNC:10474]	273670	1378510
283	ZBTB8OS	zinc finger and BTB domain containing 8 opposite strand [Source:HGNC Symbol;Acc:HGNC:24094]	57658333	200886667
284	RPLP2	ribosomal protein lateral stalk subunit P2 [Source:HGNC Symbol;Acc:HGNC:10377]	7520750	50773333
285	GBA	glucosylceramidase beta [Source:HGNC Symbol;Acc:HGNC:4177]	0	233527
286	PLEC	plectin [Source:HGNC Symbol;Acc:HGNC:9069]	36023	1419100
287	CALR	calreticulin [Source:HGNC Symbol;Acc:HGNC:1455]	517257	13842000
288	NQO1	NAD(P)H quinone dehydrogenase 1 [Source:HGNC Symbol;Acc:HGNC:2874]	6344900	12520667
289	NPM1	nucleophosmin 1 [Source:HGNC Symbol;Acc:HGNC:7910]	612140	7455400
290	COPG1	coatomer protein complex subunit gamma 1 [Source:HGNC Symbol;Acc:HGNC:2236]	0	258410
291	ANXA2	annexin A2 [Source:HGNC Symbol;Acc:HGNC:537]	17385333	527530000
292	RPL35A	ribosomal protein L35a [Source:HGNC Symbol;Acc:HGNC:10345]	0	34084333
293	ACTG1	actin gamma 1 [Source:HGNC Symbol;Acc:HGNC:144]	872040	1555633
294	COPB2	coatomer protein complex subunit beta 2 [Source:HGNC Symbol;Acc:HGNC:2232]	0	387147
295	TMED9	transmembrane p24 trafficking protein 9 [Source:HGNC Symbol;Acc:HGNC:24878]	0	10834233
296	RAB11B	RAB11B, member RAS oncogene family [Source:HGNC Symbol;Acc:HGNC:9761]	2712597	6662867
297	P4HB	prolyl 4-hydroxylase subunit beta [Source:HGNC Symbol;Acc:HGNC:8548]	1239580	3110000
298	PSMD13	proteasome 26S subunit, non-ATPase 13 [Source:HGNC Symbol;Acc:HGNC:9558]	0	705823
299	NAP1L1	nucleosome assembly protein 1 like 1 [Source:HGNC Symbol;Acc:HGNC:7637]	0	1930800
300	AKR1C1	aldo-keto reductase family 1 member C1 [Source:HGNC Symbol;Acc:HGNC:384]	7380200	22767000
301	TUBB4B	tubulin beta 4B class IVb [Source:HGNC Symbol;Acc:HGNC:20771]	4450367	18029000
302	RPL14	ribosomal protein L14 [Source:HGNC Symbol;Acc:HGNC:10305]	0	15276333

303	SF3B3	splicing factor 3b subunit 3 [Source:HGNC Symbol;Acc:HGNC:10770]	0	257333
304	AKR1C3	aldo-keto reductase family 1 member C3 [Source:HGNC Symbol;Acc:HGNC:386]	3644167	5033100
305	S100A4	S100 calcium binding protein A4 [Source:HGNC Symbol;Acc:HGNC:10494]	0	1334200
306	TUBB	tubulin beta class I [Source:HGNC Symbol;Acc:HGNC:20778]	1436800	7535633
307	PPIA	peptidylprolyl isomerase A [Source:HGNC Symbol;Acc:HGNC:9253]	52122333	80472333
308	IARS	isoleucyl-tRNA synthetase [Source:HGNC Symbol;Acc:HGNC:5330]	0	305950
309	XRCC6	X-ray repair cross complementing 6 [Source:HGNC Symbol;Acc:HGNC:4055]	0	2000033
310	NACA	nascent polypeptide associated complex subunit alpha [Source:HGNC Symbol;Acc:HGNC:7629]	0	81806
311	FLNA	filamin A [Source:HGNC Symbol;Acc:HGNC:3754]	291957	2718967
312	ANXA4	annexin A4 [Source:HGNC Symbol;Acc:HGNC:542]	533625	26233667
313	PCBP2	poly(rC) binding protein 2 [Source:HGNC Symbol;Acc:HGNC:8648]	108850	322930
314	HIST1H3C	histone cluster 1 H3 family member c [Source:HGNC Symbol;Acc:HGNC:4768]	29258000	72165000
315	PSMD12	proteasome 26S subunit, non-ATPase 12 [Source:HGNC Symbol;Acc:HGNC:9557]	0	627957
316	HNRNPAB	heterogeneous nuclear ribonucleoprotein A/B [Source:HGNC Symbol;Acc:HGNC:5034]	0	935953
317	SPTAN1	spectrin alpha, non-erythrocytic 1 [Source:HGNC Symbol;Acc:HGNC:11273]	0	160730
318	RPS26	ribosomal protein S26 [Source:HGNC Symbol;Acc:HGNC:10414]	0	2839467
319	S100A6	S100 calcium binding protein A6 [Source:HGNC Symbol;Acc:HGNC:10496]	45696667	120047333
320	RPL12	ribosomal protein L12 [Source:HGNC Symbol;Acc:HGNC:10302]	652880	10699667
321	RPS4X	ribosomal protein S4 X-linked [Source:HGNC Symbol;Acc:HGNC:10424]	793250	6843433
322	RPL23A	ribosomal protein L23a [Source:HGNC Symbol;Acc:HGNC:10317]	0	2650833
323	ASPH	aspartate beta-hydroxylase [Source:HGNC Symbol;Acc:HGNC:757]	206650	1621500
324	RPL10A	ribosomal protein L10a [Source:HGNC Symbol;Acc:HGNC:10299]	602710	34487333
325	CSNK2B	casein kinase 2 beta [Source:HGNC Symbol;Acc:HGNC:2460]	0	2518767

326	RACK1	receptor for activated C kinase 1 [Source:HGNC Symbol;Acc:HGNC:4399]	426550	19462667
327	TSN	translin [Source:HGNC Symbol;Acc:HGNC:12379]	464400	768913
328	VDAC1	voltage dependent anion channel 1 [Source:HGNC Symbol;Acc:HGNC:12669]	0	1399000
329	CLIC1	chloride intracellular channel 1 [Source:HGNC Symbol;Acc:HGNC:2062]	1747993	6609867
330	DDX3X	DEAD-box helicase 3 X-linked [Source:HGNC Symbol;Acc:HGNC:2745]	0	205968
331	RPL17	ribosomal protein L17 [Source:HGNC Symbol;Acc:HGNC:10307]	0	14982667
332	RPS18	ribosomal protein S18 [Source:HGNC Symbol;Acc:HGNC:10401]	0	57382333
333	HLA-B	major histocompatibility complex, class I, B [Source:HGNC Symbol;Acc:HGNC:4932]	0	1953233
334	PRKDC	protein kinase, DNA-activated, catalytic subunit [Source:HGNC Symbol;Acc:HGNC:9413]	0	159603
335	LYN	LYN proto-oncogene, Src family tyrosine kinase [Source:HGNC Symbol;Acc:HGNC:6735]	0	245390
336	EEF1G	eukaryotic translation elongation factor 1 gamma [Source:HGNC Symbol;Acc:HGNC:3213]	88099	616403
337	TUBB3	tubulin beta 3 class III [Source:HGNC Symbol;Acc:HGNC:20772]	201090	872907
338	AC245033.1	novel protein	0	3478933
339	TUBB8	tubulin beta 8 class VIII [Source:HGNC Symbol;Acc:HGNC:20773]	494360	1038987

Table S2. 165 highly expressed proteins in CRexo identified by LC-MS/MS

No.	Symbol	Description	Average iBAQ of CRexo	Average iBAQ of hCRexo
1	CFH	complement factor H [Source:HGNC Symbol;Acc:HGNC:4883]	12165667	262146
2	FUCA2	alpha-L-fucosidase 2 [Source:HGNC Symbol;Acc:HGNC:4008]	881037	0
3	CNTN1	contactin 1 [Source:HGNC Symbol;Acc:HGNC:2171]	5812133	2541933
4	TMSB10	thymosin beta 10 [Source:HGNC Symbol;Acc:HGNC:11879]	35076667	8123500
5	TIMP2	TIMP metallopeptidase inhibitor 2 [Source:HGNC Symbol;Acc:HGNC:11821]	1801467	635020
6	VCAN	versican [Source:HGNC Symbol;Acc:HGNC:2464]	96951333	27031000
7	CP	ceruloplasmin [Source:HGNC Symbol;Acc:HGNC:2295]	14283000	11334367
8	HEXB	hexosaminidase subunit beta [Source:HGNC Symbol;Acc:HGNC:4879]	15560333	3676200
9	DKK3	dickkopf WNT signaling pathway inhibitor 3 [Source:HGNC Symbol;Acc:HGNC:2893]	1570037	273055
10	ITIH1	inter-alpha-trypsin inhibitor heavy chain 1 [Source:HGNC Symbol;Acc:HGNC:6166]	424297	0
11	GPC1	glypican 1 [Source:HGNC Symbol;Acc:HGNC:4449]	7239333	2394800
12	NTN4	netrin 4 [Source:HGNC Symbol;Acc:HGNC:13658]	1629767	421550
13	FBLN1	fibulin 1 [Source:HGNC Symbol;Acc:HGNC:3600]	38101333	6755833
14	APOB	apolipoprotein B [Source:HGNC Symbol;Acc:HGNC:603]	3334833	955703
15	EPDR1	ependymin related 1 [Source:HGNC Symbol;Acc:HGNC:17572]	3099800	0
16	NID2	nidogen 2 [Source:HGNC Symbol;Acc:HGNC:13389]	27208667	9912800
17	ATRN	attractin [Source:HGNC Symbol;Acc:HGNC:885]	3242567	362190
18	F11	coagulation factor XI [Source:HGNC Symbol;Acc:HGNC:3529]	252130	0
19	NRCAM	neuronal cell adhesion molecule [Source:HGNC Symbol;Acc:HGNC:7994]	189280	0
20	LAMB1	laminin subunit beta 1 [Source:HGNC Symbol;Acc:HGNC:6486]	93096000	36400667
21	TF	transferrin [Source:HGNC Symbol;Acc:HGNC:11740]	15113000	43858
22	NRP1	neuropilin 1 [Source:HGNC Symbol;Acc:HGNC:8004]	2262267	705257
23	ARSA	arylsulfatase A [Source:HGNC Symbol;Acc:HGNC:713]	956823	0
24	PROCR	protein C receptor [Source:HGNC Symbol;Acc:HGNC:9452]	2351033	0
25	CHMP4B	charged multivesicular body protein 4B [Source:HGNC Symbol;Acc:HGNC:16171]	3468033	1601267
26	CST3	cystatin C [Source:HGNC Symbol;Acc:HGNC:2475]	48224333	14374000
27	TIMP1	TIMP metallopeptidase inhibitor 1 [Source:HGNC Symbol;Acc:HGNC:11820]	31039000	12242867

28	GLA	galactosidase alpha [Source:HGNC Symbol;Acc:HGNC:4296]	1250800	338580
29	NUCB1	nucleobindin 1 [Source:HGNC Symbol;Acc:HGNC:8043]	7620800	714747
30	DNASE2	deoxyribonuclease 2, lysosomal [Source:HGNC Symbol;Acc:HGNC:2960]	2323267	646800
31	C5	complement C5 [Source:HGNC Symbol;Acc:HGNC:1331]	2009567	371423
32	AMBP	alpha-1-microglobulin/bikunin precursor [Source:HGNC Symbol;Acc:HGNC:453]	2768000	0
33	DKK1	dickkopf WNT signaling pathway inhibitor 1 [Source:HGNC Symbol;Acc:HGNC:2891]	2781467	0
34	TASOR2	transcription activation suppressor family member 2 [Source:HGNC Symbol;Acc:HGNC:23484]	2150533	1031500
35	LGALS3BP	galectin 3 binding protein [Source:HGNC Symbol;Acc:HGNC:6564]	924030000	357420000
36	NAGLU	N-acetyl-alpha-glucosaminidase [Source:HGNC Symbol;Acc:HGNC:7632]	915280	156794
37	VTN	vitronectin [Source:HGNC Symbol;Acc:HGNC:12724]	3488233	930797
38	CTSC	cathepsin C [Source:HGNC Symbol;Acc:HGNC:2528]	12400333	681653
39	SIAE	sialic acid acetylesterase [Source:HGNC Symbol;Acc:HGNC:18187]	649893	0
40	HPX	hemopexin [Source:HGNC Symbol;Acc:HGNC:5171]	5022633	518335
41	APOC3	apolipoprotein C3 [Source:HGNC Symbol;Acc:HGNC:610]	20382333	2716800
42	AC006064.6	novel transcript	1711067	522000
43	SOD2	superoxide dismutase 2 [Source:NCBI gene;Acc:6648]	8493100	748527
44	VNN1	vanin 1 [Source:HGNC Symbol;Acc:HGNC:12705]	1664267	0
45	ADGRG6	adhesion G protein-coupled receptor G6 [Source:HGNC Symbol;Acc:HGNC:13841]	1224597	338470
46	KNG1	kininogen 1 [Source:HGNC Symbol;Acc:HGNC:6383]	2651233	186180
47	HRG	histidine rich glycoprotein [Source:HGNC Symbol;Acc:HGNC:5181]	2879333	358450
48	COL7A1	collagen type VII alpha 1 chain [Source:HGNC Symbol;Acc:HGNC:2214]	962667	127491
49	FN1	fibronectin 1 [Source:HGNC Symbol;Acc:HGNC:3778]	101647667	41376000
50	QPCT	glutaminyl-peptide cyclotransferase [Source:HGNC Symbol;Acc:HGNC:9753]	850080	0
51	NID1	nidogen 1 [Source:HGNC Symbol;Acc:HGNC:7821]	304480	0
52	SERPINC1	serpin family C member 1 [Source:HGNC Symbol;Acc:HGNC:775]	142268	0
53	MUC5B	mucin 5B, oligomeric mucus/gel-forming [Source:HGNC Symbol;Acc:HGNC:7516]	571256667	374660000
54	CTSD	cathepsin D [Source:HGNC Symbol;Acc:HGNC:2529]	42950667	23813667
55	APOA1	apolipoprotein A1 [Source:HGNC Symbol;Acc:HGNC:600]	53155333	13352333
56	TTR	transthyretin [Source:HGNC Symbol;Acc:HGNC:12405]	4255867	0

57	RARRES1	retinoic acid receptor responder 1 [Source:HGNC Symbol;Acc:HGNC:9867]	711980	0
58	TGFBI	transforming growth factor beta induced [Source:HGNC Symbol;Acc:HGNC:11771]	35665667	19026000
59	CLU	clusterin [Source:HGNC Symbol;Acc:HGNC:2095]	197910000	94098667
60	PLG	plasminogen [Source:HGNC Symbol;Acc:HGNC:9071]	10606767	220140
61	SRGN	serglycin [Source:HGNC Symbol;Acc:HGNC:9361]	73280000	12658000
62	C4BPA	complement component 4 binding protein alpha [Source:HGNC Symbol;Acc:HGNC:1325]	9359400	1724637
63	C3	complement C3 [Source:HGNC Symbol;Acc:HGNC:1318]	59286333	15950667
64	LDLR	low density lipoprotein receptor [Source:HGNC Symbol;Acc:HGNC:6547]	1736300	474923
65	APOE	apolipoprotein E [Source:HGNC Symbol;Acc:HGNC:613]	7421667	5027167
66	APOC1	apolipoprotein C1 [Source:HGNC Symbol;Acc:HGNC:607]	1984767	0
67	LAMA5	laminin subunit alpha 5 [Source:HGNC Symbol;Acc:HGNC:6485]	85247667	33197333
68	LOXL2	lysyl oxidase like 2 [Source:HGNC Symbol;Acc:HGNC:6666]	38071333	15730333
69	COL4A2	collagen type IV alpha 2 chain [Source:HGNC Symbol;Acc:HGNC:2203]	427040	154876
70	CTSL	cathepsin L [Source:HGNC Symbol;Acc:HGNC:2537]	145953333	27734667
71	GOLM1	golgi membrane protein 1 [Source:HGNC Symbol;Acc:HGNC:15451]	10679567	2398000
72	NT5E	5'-nucleotidase ecto [Source:HGNC Symbol;Acc:HGNC:8021]	1706767	840727
73	GNS	glucosamine (N-acetyl)-6-sulfatase [Source:HGNC Symbol;Acc:HGNC:4422]	6584200	2032433
74	LAMC1	laminin subunit gamma 1 [Source:HGNC Symbol;Acc:HGNC:6492]	90559000	38668333
75	TXN	thioredoxin [Source:HGNC Symbol;Acc:HGNC:12435]	29696000	20029333
76	TLN1	talin 1 [Source:HGNC Symbol;Acc:HGNC:11845]	74274	45297
77	RIPK1	receptor interacting serine/threonine kinase 1 [Source:HGNC Symbol;Acc:HGNC:10019]	805890	0
78	GGH	gamma-glutamyl hydrolase [Source:HGNC Symbol;Acc:HGNC:4248]	20367000	1946800
79	SDCBP	syndecan binding protein [Source:HGNC Symbol;Acc:HGNC:10662]	267280000	221446667
80	THBS1	thrombospondin 1 [Source:HGNC Symbol;Acc:HGNC:11785]	6927967	3135733
81	MFGE8	milk fat globule-EGF factor 8 protein [Source:HGNC Symbol;Acc:HGNC:7036]	10610667	3028967
82	GALNS	galactosamine (N-acetyl)-6-sulfatase [Source:HGNC Symbol;Acc:HGNC:4122]	3366533	1015360
83	IGFBP4	insulin like growth factor binding protein 4 [Source:HGNC Symbol;Acc:HGNC:5473]	59994000	8749533

84	RCN3	reticulocalbin 3 [Source:HGNC Symbol;Acc:HGNC:21145]	507403	0
85	HSPG2	heparan sulfate proteoglycan 2 [Source:HGNC Symbol;Acc:HGNC:5273]	74905000	19619667
86	GC	GC vitamin D binding protein [Source:HGNC Symbol;Acc:HGNC:4187]	963827	0
87	LCN2	lipocalin 2 [Source:HGNC Symbol;Acc:HGNC:6526]	30990000	3981433
88	PRSS23	serine protease 23 [Source:HGNC Symbol;Acc:HGNC:14370]	14090300	3877633
89	CRIM1	cysteine rich transmembrane BMP regulator 1 [Source:HGNC Symbol;Acc:HGNC:2359]	589970	0
90	UCHL1	ubiquitin C-terminal hydrolase L1 [Source:HGNC Symbol;Acc:HGNC:12513]	7781333	2579300
91	LRP8	LDL receptor related protein 8 [Source:HGNC Symbol;Acc:HGNC:6700]	796303	0
92	APOA2	apolipoprotein A2 [Source:HGNC Symbol;Acc:HGNC:601]	6136867	2766833
93	C1QC	complement C1q C chain [Source:HGNC Symbol;Acc:HGNC:1245]	3504800	786270
94	C1R	complement C1r [Source:HGNC Symbol;Acc:HGNC:1246]	19435667	2174167
95	IGFBP7	insulin like growth factor binding protein 7 [Source:HGNC Symbol;Acc:HGNC:5476]	929573	0
96	COL1A2	collagen type I alpha 2 chain [Source:HGNC Symbol;Acc:HGNC:2198]	296483	121480
97	CTSB	cathepsin B [Source:HGNC Symbol;Acc:HGNC:2527]	39705667	7761033
98	CLDN2	claudin 2 [Source:HGNC Symbol;Acc:HGNC:2041]	3103100	947300
99	TPP1	tripeptidyl peptidase 1 [Source:HGNC Symbol;Acc:HGNC:2073]	4367367	589517
100	B2M	beta-2-microglobulin [Source:HGNC Symbol;Acc:HGNC:914]	21816000<br>0	45414667
101	IGFBP6	insulin like growth factor binding protein 6 [Source:HGNC Symbol;Acc:HGNC:5475]	3854667	0
102	BMP1	bone morphogenetic protein 1 [Source:HGNC Symbol;Acc:HGNC:1067]	889180	193940
103	ADAM9	ADAM metallopeptidase domain 9 [Source:HGNC Symbol;Acc:HGNC:216]	677583	143450
104	PCSK9	proprotein convertase subtilisin/kexin type 9 [Source:HGNC Symbol;Acc:HGNC:20001]	684010	283700
105	BTD	biotinidase [Source:HGNC Symbol;Acc:HGNC:1122]	2674167	391550
106	GLB1	galactosidase beta 1 [Source:HGNC Symbol;Acc:HGNC:4298]	1452767	730577
107	CST1	cystatin SN [Source:HGNC Symbol;Acc:HGNC:2473]	22494000	0
108	CDH2	cadherin 2 [Source:HGNC Symbol;Acc:HGNC:1759]	251587	0
109	PDGFD	platelet derived growth factor D [Source:HGNC Symbol;Acc:HGNC:30620]	622133	0
110	FGG	fibrinogen gamma chain [Source:HGNC Symbol;Acc:HGNC:3694]	22290333<br>3	633517

111	FGA	fibrinogen alpha chain [Source:HGNC Symbol;Acc:HGNC:3661]	144910000	1258787
112	FGB	fibrinogen beta chain [Source:HGNC Symbol;Acc:HGNC:3662]	235970000	3820533
113	CLSTN1	calsyntenin 1 [Source:HGNC Symbol;Acc:HGNC:17447]	9792733	1767933
114	PRNP	prion protein [Source:HGNC Symbol;Acc:HGNC:9449]	7336033	986110
115	LAMB2	laminin subunit beta 2 [Source:HGNC Symbol;Acc:HGNC:6487]	338513	111825
116	C1QB	complement C1q B chain [Source:HGNC Symbol;Acc:HGNC:1242]	2410767	826820
117	A2M	alpha-2-macroglobulin [Source:HGNC Symbol;Acc:HGNC:7]	42296333	144850
118	CD151	CD151 molecule (Raph blood group) [Source:HGNC Symbol;Acc:HGNC:1630]	5620900	1425800
119	FUCA1	alpha-L-fucosidase 1 [Source:HGNC Symbol;Acc:HGNC:4006]	1235267	0
120	HIST1H2AJ	histone cluster 1 H2A family member j [Source:HGNC Symbol;Acc:HGNC:4727]	50013000	22165000
121	ATP6AP2	ATPase H+ transporting accessory protein 2 [Source:HGNC Symbol;Acc:HGNC:18305]	1271997	0
122	C1S	complement C1s [Source:HGNC Symbol;Acc:HGNC:1247]	16114667	2278333
123	TSKU	tsukushi, small leucine rich proteoglycan [Source:HGNC Symbol;Acc:HGNC:28850]	7355933	3985133
124	PAPPA	pappalysin 1 [Source:HGNC Symbol;Acc:HGNC:8602]	6161133	1666867
125	COL18A1	collagen type XVIII alpha 1 chain [Source:HGNC Symbol;Acc:HGNC:2195]	10337533	2198167
126	AGRN	agrin [Source:HGNC Symbol;Acc:HGNC:329]	185756667	54726000
127	HBA1	hemoglobin subunit alpha 1 [Source:HGNC Symbol;Acc:HGNC:4823]	1527733	0
128	PSAP	prosaposin [Source:HGNC Symbol;Acc:HGNC:9498]	40138333	24160000
129	CFD	complement factor D [Source:HGNC Symbol;Acc:HGNC:2771]	1498767	0
130	F5	coagulation factor V [Source:HGNC Symbol;Acc:HGNC:3542]	68560	0
131	L1CAM	L1 cell adhesion molecule [Source:HGNC Symbol;Acc:HGNC:6470]	87416	0
132	COL5A2	collagen type V alpha 2 chain [Source:HGNC Symbol;Acc:HGNC:2210]	3882733	505510
133	NEU1	neuraminidase 1 [Source:HGNC Symbol;Acc:HGNC:7758]	325797	0
134	ADGRG1	adhesion G protein-coupled receptor G1 [Source:HGNC Symbol;Acc:HGNC:4512]	1659200	0
135	TMSB4X	thymosin beta 4 X-linked [Source:HGNC Symbol;Acc:HGNC:11881]	54850333	14981000

136	IGKC	immunoglobulin kappa constant [Source:HGNC Symbol;Acc:HGNC:5716]	1202366667	205373333
137	IGKV3-11	immunoglobulin kappa variable 3-11 [Source:HGNC Symbol;Acc:HGNC:5815]	8981600	0
138	IGLV1-47	immunoglobulin lambda variable 1-47 [Source:HGNC Symbol;Acc:HGNC:5880]	12015333	3705467
139	IGLV3-25	immunoglobulin lambda variable 3-25 [Source:HGNC Symbol;Acc:HGNC:5908]	8894467	0
140	IGLC3	immunoglobulin lambda constant 3 (Kern-Oz+ marker) [Source:HGNC Symbol;Acc:HGNC:5857]	40371333	11643100
141	IGHG2	immunoglobulin heavy constant gamma 2 (G2m marker) [Source:HGNC Symbol;Acc:HGNC:5526]	153010000	25655000
142	IGHA1	immunoglobulin heavy constant alpha 1 [Source:HGNC Symbol;Acc:HGNC:5478]	78157000	2816500
143	IGHG1	immunoglobulin heavy constant gamma 1 (G1m marker) [Source:HGNC Symbol;Acc:HGNC:5525]	747873333	80825000
144	IGHG3	immunoglobulin heavy constant gamma 3 (G3m marker) [Source:HGNC Symbol;Acc:HGNC:5527]	64224333	2832133
145	IGHM	immunoglobulin heavy constant mu [Source:HGNC Symbol;Acc:HGNC:5541]	84075667	32516667
146	IGHV3-7	immunoglobulin heavy variable 3-7 [Source:HGNC Symbol;Acc:HGNC:5620]	4334500	0
147	IGHV4-59	immunoglobulin heavy variable 4-59 [Source:HGNC Symbol;Acc:HGNC:5654]	7087033	0
148	IGHV5-51	immunoglobulin heavy variable 5-51 [Source:HGNC Symbol;Acc:HGNC:5659]	929750	0
149	HEXA	hexosaminidase subunit alpha [Source:HGNC Symbol;Acc:HGNC:4878]	10830333	2819767
150	APOC2	apolipoprotein C2 [Source:HGNC Symbol;Acc:HGNC:609]	4821633	0
151	ORM2	orosomucoid 2 [Source:HGNC Symbol;Acc:HGNC:8499]	1159867	0
152	IGKV3-15	immunoglobulin kappa variable 3-15 [Source:HGNC Symbol;Acc:HGNC:5816]	12649333	0
153	ORM1	orosomucoid 1 [Source:HGNC Symbol;Acc:HGNC:8498]	3974467	468850
154	IGKV1D-12	immunoglobulin kappa variable 1D-12 [Source:HGNC Symbol;Acc:HGNC:5746]	3741900	0
155	IGKV1-33	immunoglobulin kappa variable 1-33 [Source:HGNC Symbol;Acc:HGNC:5737]	2558567	0
156	CFB	complement factor B [Source:HGNC Symbol;Acc:HGNC:1037]	2138233	612130
157	C4A	complement C4A (Rodgers blood group) [Source:HGNC Symbol;Acc:HGNC:1323]	9177533	5917933
158	HBB	hemoglobin subunit beta [Source:HGNC Symbol;Acc:HGNC:4827]	6515933	2230833

159	IGLL5	immunoglobulin lambda like polypeptide 5 [Source:HGNC Symbol;Acc:HGNC:38476]	551020000	107026667
160	CHMP1B	charged multivesicular body protein 1B [Source:HGNC Symbol;Acc:HGNC:24287]	4493433	0
161	HP	haptoglobin [Source:HGNC Symbol;Acc:HGNC:5141]	59593333	2202027
162	IGHV3OR16-12	immunoglobulin heavy variable 3/OR16-12 (non-functional) [Source:HGNC Symbol;Acc:HGNC:5636]	4212533	0
163	IGHV3OR16-9	immunoglobulin heavy variable 3/OR16-9 (non-functional) [Source:HGNC Symbol;Acc:HGNC:5644]	36471000	7371367
164	AC135068.8	novel gene identicle to IGHV1OR15-1	6852633	0
165	FCGBP	Fc fragment of IgG binding protein [Source:HGNC Symbol;Acc:HGNC:13572]	69619333	50605000
